# Cost-effectiveness analysis of interventions to improve diagnosis and preventive therapy for paediatric tuberculosis in 9 sub-Saharan African countries: A modelling study

**DOI:** 10.1371/journal.pmed.1004285

**Published:** 2023-09-06

**Authors:** Nyashadzaishe Mafirakureva, Sushant Mukherjee, Mikhael de Souza, Cassandra Kelly-Cirino, Mario J. P. Songane, Jennifer Cohn, Jean-François Lemaire, Martina Casenghi, Peter J. Dodd

**Affiliations:** 1 Sheffield Centre for Health and Related Research, University of Sheffield, Sheffield, United Kingdom; 2 Elizabeth Glaser Pediatric AIDS Foundation (EGPAF), Washington, DC, United States of America; 3 Division of Infectious Diseases, University of Pennsylvania School of Medicine, Philadelphia, Pennsylvania, United States of America

## Abstract

**Background:**

Over 1 million children aged 0 to 14 years were estimated to develop tuberculosis in 2021, resulting in over 200,000 deaths. Practical interventions are urgently needed to improve diagnosis and antituberculosis treatment (ATT) initiation in children aged 0 to 14 years and to increase coverage of tuberculosis preventive therapy (TPT) in children at high risk of developing tuberculosis disease. The multicountry CaP-TB intervention scaled up facility-based intensified case finding and strengthened household contact management and TPT provision at HIV clinics. To add to the limited health-economic evidence on interventions to improve ATT and TPT in children, we evaluated the cost-effectiveness of the CaP-TB intervention.

**Methods and findings:**

We analysed clinic-level pre/post data to quantify the impact of the CaP-TB intervention on ATT and TPT initiation across 9 sub-Saharan African countries. Data on tuberculosis diagnosis and ATT/TPT initiation counts with corresponding follow-up time were available for 146 sites across the 9 countries prior to and post project implementation, stratified by 0 to 4 and 5 to 14 year age-groups. Preintervention data were retrospectively collected from facility registers for a 12-month period, and intervention data were prospectively collected from December 2018 to June 2021 using project-specific forms. Bayesian generalised linear mixed-effects models were used to estimate country-level rate ratios for tuberculosis diagnosis and ATT/TPT initiation. We analysed project expenditure and cascade data to determine unit costs of intervention components and used mathematical modelling to project health impact, health system costs, and cost-effectiveness.

Overall, ATT and TPT initiation increased, with country-level incidence rate ratios varying between 0.8 (95% uncertainty interval [UI], 0.7 to 1.0) and 2.9 (95% UI, 2.3 to 3.6) for ATT and between 1.6 (95% UI, 1.5 to 1.8) and 9.8 (95% UI, 8.1 to 11.8) for TPT. We projected that for every 100 children starting either ATT or TPT at baseline, the intervention package translated to between 1 (95% UI, −1 to 3) and 38 (95% UI, 24 to 58) deaths averted, with a median incremental cost-effectiveness ratio (ICER) of US$634 per disability-adjusted life year (DALY) averted. ICERs ranged between US$135/DALY averted in Democratic of the Congo and US$6,804/DALY averted in Cameroon. The main limitation of our study is that the impact is based on pre/post comparisons, which could be confounded.

**Conclusions:**

In most countries, the CaP-TB intervention package improved tuberculosis treatment and prevention services for children aged under 15 years, but large variation in estimated impact and ICERs highlights the importance of local context.

**Trial registration:**

This evaluation is part of the TIPPI study, registered with ClinicalTrials.gov (NCT03948698).

## Abstract 2

In a modelling study, Nyashadzaishe Mafirakureva and colleagues, examine the cost-effectiveness of health interventions designed to improve tuberculosis prevention and treatment in children.

## Author summary

**Why was this study done?:**

- Tuberculosis in children under 15 years of age is a substantial cause of disease and death.
- There is very limited evidence on the cost and cost-effectiveness of interventions to improve tuberculosis prevention and treatment in children.

**What did the researchers do and find?:**

- We analysed financial and cascade data from a health system strengthening intervention (CaP-TB) to improve tuberculosis case finding, treatment, and provision of tuberculosis preventive treatment across 9 sub-Saharan African countries.
- We modelled the health impact, resource use, and costs of the intervention to estimate incremental cost-effectiveness ratios (ICERs) relative to the standard of care (SoC).
- Household contact management and provision of tuberculosis preventive treatment was typically more cost-effective than the tuberculosis case finding and treatment component but made a smaller contribution to saving lives.
- Across the 9 countries, we found that for every 100 children who started either antituberculosis treatment or tuberculosis preventive therapy under SoC, a median of 7 deaths were averted, and we estimated a median ICER of US$634 per disability-adjusted life years averted.

**What do these findings mean?:**

- The CaP-TB intervention generally increased tuberculosis case finding, antituberculosis treatment, and tuberculosis preventive treatment for children aged under 15 years, which was estimated to avert tuberculosis and save lives.
- There was a large variation in impact, costs, and ICERs across countries, highlighting the importance of local context.
- The main limitation of our analysis is that our measure of effect is based on pre/post comparisons, rather than data from a randomised study, leaving it vulnerable to trends or shocks and lacking true causal attribution.

## Introduction

Over the last decade, there has been an increasing realisation that tuberculosis in children under 15 years of age is a substantial cause of disease and death [1]. The World Health Organization (WHO) estimated 1 million children developed tuberculosis and 230,000 died in 2020 [2], making tuberculosis a top 10 cause of death in children under 5 years [3]. Diagnosis of tuberculosis in children is complicated by often paucibacillary disease, difficulty in obtaining diagnostic samples, and nonspecific symptoms [4–6]; the majority of deaths are estimated to occur in children whose diagnosis has been missed [1]. Children are at high risk of developing tuberculosis following household exposure [7], and WHO recommends household contact management (HHCM) to find tuberculosis among children living with newly diagnosed adults and provide tuberculosis preventive therapy (TPT) to children without tuberculosis disease to reduce their chances of progressing [8]. Modelling suggests HHCM could have a substantial impact on paediatric tuberculosis [9], but coverage remains low [10].

Establishing practical approaches to improve routine detection and antituberculosis treatment (ATT) for children with tuberculosis and increased use of HHCM and TPT is of central importance. The CaP-TB intervention package, which was implemented in 9 sub-Saharan African countries, included facility-based intensified case finding (ICF) to improve detection, community-based HHCM, and strengthened provision of TPT to children living with HIV at HIV clinics.

In 2022, WHO released updated guidelines on tuberculosis management in children and adolescents [11] and an operational handbook documenting experience with different interventions, which includes additional details of the CaP-TB intervention [11]. Evaluating the impact of interventions on health outcomes and establishing their cost-effectiveness is a key piece of evidence for policy makers considering their adoption. As part of the WHO guideline development process [11], a systematic review was conducted to identify evidence on models of care for paediatric tuberculosis case detection and TPT. This review identified 16 studies across all subquestions, none of which had an associated economic evaluation. A PubMed search for economic evaluations related to paediatric tuberculosis (on 15 July 2022) yielded only 3 studies reporting incremental cost-effectiveness ratios (ICERs) for tuberculosis case finding [12–14] and 3 reporting ICERs for TPT interventions [15–17]. However, these studies were based on hypothetical rather than implemented interventions.

In order to address the lack of health economic evidence for interventions introduced to improve paediatric ATT and TPT provision, we undertook an economic evaluation of the CaP-TB intervention. We use data from before and after the implementation of the CaP-TB strategy in 9 sub-Saharan African countries to quantify for each country the changes in ATT and TPT provision, to model the impact of these interventions on mortality and health system costs, and to estimate their cost-effectiveness.

## Methods

We undertook a health systems perspective cost-utility analysis of the CaP-TB intervention for children aged under 15 years against a standard of care (SoC) in Cameroon, Côte d’Ivoire, Democratic Republic of the Congo (DRC), Kenya, Lesotho, Malawi, Uganda, Tanzania, and Zimbabwe. We used a statistical analysis of pre/post rate data to determine changes in resource use in each country and extended this with mathematical modelling to capture impacts on mortality. All analyses used probabilistic sensitivity analysis and were stratified by ages 0 to 4 years and 5 to 14 years and HIV. We considered the ICF, and HHCM and HIV clinic TPT aspects of the intervention separately, and also as a combined intervention package. All analysis code and data are available on GitHub.

### Ethics statement

Ethical approval was granted by Advarra (Pro00028743), the WHO Ethics Review Committee (ERC), and institutional review boards in each country. A waiver for informed consent was granted for patient-level data collection by Advarra, WHO ERC, and country institutional review boards. This evaluation is part of the TIPPI study, registered with ClinicalTrials.gov (NCT03948698).

### Description of intervention

Details of the CaP-TB intervention and the pre/post study design including data collection are described elsewhere, including the WHO operational handbook [11]. The facility-based ICF approach developed employed a child-adapted tuberculosis symptom-based screening tool at different entry points attended by children (outpatient department, inpatient departments, HIV clinics, maternal and children health clinics, and nutrition clinics), often with support from community healthcare workers (CHWs). Sample collection procedures were strengthened, and access to chest X-ray and Xpert MTB/RIF testing as an initial diagnostic increased. HHCM used community-based rather than facility-based contact screening where possible, with referral to facilities for symptom evaluation or TPT initiation in asymptomatic children aged under 5 years and over 5 years living with HIV. The 3RH TPT regimen was adopted in countries allowing its use. Provision of TPT to children living with HIV at HIV clinics was also strengthened. For evaluation purposes, a subset of sites in each country were selected and data on ATT and TPT initiation before and after the intervention start were recorded.

The SoC was generally characterised by low coverage of paediatric tuberculosis services (tuberculosis diagnostic and treatment and especially TPT) but varied widely across countries in terms of paediatric tuberculosis diagnostic capacity, decentralisation, and integration of paediatric tuberculosis services. This variation was a reflection of the different geographical, epidemiological, health system, policy/operational and economic contexts in these settings. As a result, the CaP-TB intervention was customised for each country based on existing (SoC) national policies and guidelines, planned and existing activities supported through other efforts, and country-specific paediatric TB burden and barriers and bottlenecks.

The pre/post project (quasi-experimental) intervention evaluation included all paediatric patients (0 to 14 years) presenting and receiving tuberculosis services (including screening, diagnosis and treatment for active TB, and provision of TPT) in CaP-TB project sites across the 9 sub-Saharan African countries prior to and during project implementation. Preintervention data were retrospectively collected from appropriate MoH registers (e.g., presumptive TB, TB treatment, IPT, and contact investigation) for all entries recorded over a complete 12-month period ending at least 6 months before the date of data extraction. The preintervention data collection period varied by country but generally ranged between March 2017 and August 2018. Intervention data were prospectively collected for the period running December 2018 to June 2021 using project-specific screening, investigation, and treatment forms. The before and after comparison/analysis was based on data from 144 sites enrolled in the CaP-TB project for which preintervention data could be collected.

### Analysis of intervention effect

We used count data for each site on tuberculosis diagnoses, ATT initiations, and TPT initiations during a preintervention observation period, and after the intervention was implemented (within calendar period December 2018 to December 2020). We calculated median rates pre/post intervention and analysed these data with a generalised linear mixed-effects model that estimated site- and country-level rate ratios for tuberculosis diagnosis, ATT initiation, and TPT initiation. This model was implemented in a Bayesian framework fitted using Markov chain Monte Carlo (see S1 Appendix), and a sample of 10,000 draws from the posterior country-level rate ratios was used as the basis for a probabilistic sensitivity analysis of incremental costs and effects. We did not adjust for any variables.

### Modelling of cascades and outcomes

**Fig 1:**
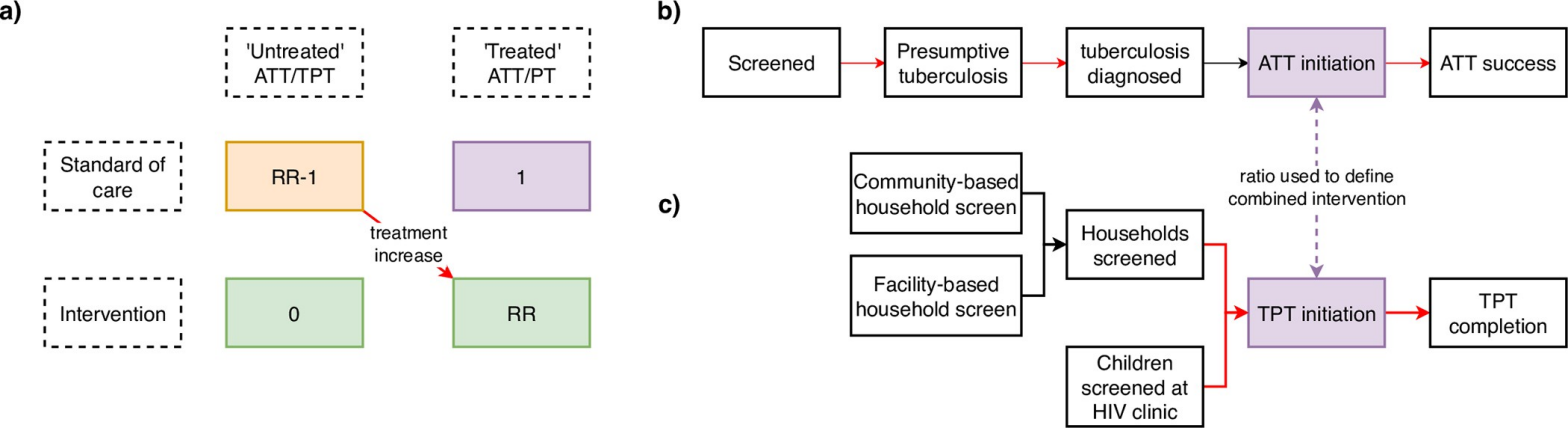
Conceptual diagram for the mathematical model used to project health impact, health system costs, and cost-effectiveness of the CaP-TB intervention on ATT and TPT initiation across 9 sub-Saharan African countries in comparison to SoC. Red lines indicate changes under intervention. Panel (**a**) illustrates how RR summaries are used to model changes in ATT/TPT initiation coverage in a hypothetical cohort. Panel (**b**) describes the cascade steps for ATT. Panel (**c**) describes the cascade for TPT. The combined intervention is defined by a ratio between initiation boxes (purple) in panels (**b**) and (**c**), and the intervention results are described relative to the numbers initiating at baseline (purple box in panel **a**). ATT, antituberculosis treatment; CaP-TB, Catalyzing Pediatric TB Innovations; RR, risk ratio; SoC, standard of care; TPT, tuberculosis preventive therapy.

We calculated the number of ATT or TPT initiations among 0- to 4- and 5- to 14-year-old children under the intervention per initiation under SoC using country-level rate ratios. We used data on activities per ATT or TPT initiation in each cascade of care, and the intervention effect on these, to model associated resource use under SoC and intervention (see Fig 1). The combined intervention package was modelled using the relative number of ATT and TPT initiations under intervention. We used literature data from systematic review to inform risks of incident tuberculosis or tuberculosis death. Disability-adjusted life years (DALYs) were calculated using adult morbidity assumptions and country-specific life years lived, discounted at 3% per year and averaged over age categories, with 0% and 5% discount rates used in sensitivity analyses. We also generated results neglecting the contribution of morbidity to DALYs.

For the ATT cascade, we used intervention data to calculate the relative number of children screened, considered as presumptive tuberculosis, diagnosed with tuberculosis, initiated on ATT, and completing treatment. Lacking data on relative numbers screened under SoC, we assumed the number screened per child considered presumptive was unchanged by intervention. Five countries had data to define changes in the remaining cascade steps during intervention; the mean relative changes were applied to the other 4 countries.

We used systematic review estimates of case fatality ratios (CFRs) for treated and untreated children with tuberculosis, stratified by age category and HIV/ART status [1,18]. Tuberculosis deaths were assumed to occur within a year. We used baseline data on prevalence of HIV in those diagnosed with tuberculosis, and age mix by country and assumed all children with HIV were on ART.

For TPT, we used data on the number of households screened per TPT initiation, the split of community-based versus facility-based HHCM, the split of children initiating TPT at HIV clinics versus via HHCM, the number of children found with prevalent tuberculosis disease, and the number of TPT initiations completing treatment. In the absence of SoC screening data, we assumed the SoC ratio of screens per child identified presumptive tuberculosis was the same as under the intervention.

For child contacts with tuberculosis disease, their chance of being treated for tuberculosis without HHCM was based on WHO estimates of country- and age-specific case detection ratios (CDRs) but inflated to account for a higher chance of detection among children with detected index cases, as in previous work [9,17]. Risks of progression to incident tuberculosis disease in household contacts were based on systematic review data [7]. For children living with HIV, progression to incident tuberculosis was modelled assuming an annual infection risk of 1%, and inflating progression risks by an incidence rate ratio for children on ART based on systematic review [19]. Incident tuberculosis was assumed to receive ATT with the background age-specific CDR.

Additional modelling details including parameter sources and distributions are provided in S1 Appendix.

Because of a change from retrospective data collection on ATT outcomes and TPT completion under SoC to prospective data collection under the intervention, our primary analyses assumed only intervention levels of success/completion. As sensitivity analyses, we considered the impact of changes in ATT/TPT success/completion between SoC and intervention using data from Table A1 in S1 Appendix.

### Resource use and health economic outputs

To value resources use, we used a mixture of top-down, activity-based costing using CaP-TB financial records, limited bottom-up costing, and adjusted costs from literature to establish unit costs for activities under the SoC and the intervention. All costs were converted to 2020 US$ using appropriate inflation rates.

To estimate intervention costs, budget data were organised in cost categories and subcategories, excluding costs not directly relevant to project implementation and EGPAF overheads (see S1 Appendix). The contribution of each subcategory to the overall cost category was calculated and applied to EGPAF expenditure to disaggregate expenditure to the subcategory level. For tuberculosis medicines and diagnostics, exact costs incurred by each country for each item purchased were directly taken from expenditure data.

Costs were assigned to the following activity groupings: project setup and demand generation; community-based household contact tracing; facility-based household contact tracing; screening of non-contacts in HIV entry points; screening of non-contacts in non-HIV entry points; tuberculosis evaluation and diagnosis; tuberculosis treatment; tuberculosis preventive therapy; evaluation (including monitoring and evaluation and research); and programme management. Research-specific costs were excluded in the final analysis. Project country teams were asked to assess the proportion of costs for each category and subcategory that was associated with each direct patient care activity, and this was used to disaggregate costs across activities and develop unit costs for each patient activity.

Costs for SoC tuberculosis diagnosis and treatment were derived from published literature sources. Country-specific costs were used where possible. Otherwise, costs were transferred from other countries by applying relevant purchasing power parity conversion factors.

Total costs under SoC and intervention were calculated by attaching the SoC and intervention unit costs for each activity to the relevant activity driver in the model. We computed the cost per child initiated on ATT or TPT under SoC and the intervention, the incremental cost of the intervention per child initiated, and the ICER of the intervention as US$ per DALY averted, and the cost-effectiveness acceptability curves.

## Results

### Baseline rates and intervention effect

Before-after data were based on 146 sites with a total site-time of 14,266 months for ATT and 151 sites with a total site-time of 14,588 months for TPT. The overall mean ATT initiation rate was 0.81 per month per site at baseline and 1.28 per month under the intervention, with 72% of sites having a higher intervention ATT initiation rate. The overall mean TPT initiation rate was 0.89 per month at baseline and 3.61 per month under the intervention, with 91% of sites having a higher intervention TPT initiation rate. Country mean ATT success rates ranged between 49% and 88% at baseline and between 84% and 95% under the intervention. Country mean TPT completion rates ranged between 33% and 93% at baseline, and between 83% and 99% under the intervention. The proportion of children aged 0 to 14 year diagnosed with tuberculosis with bacteriological confirmation was 14.1% at baseline and 22.4% under the intervention. See Figures A1-A6 in S1 Appendix for visualisations.

**Fig 2:**
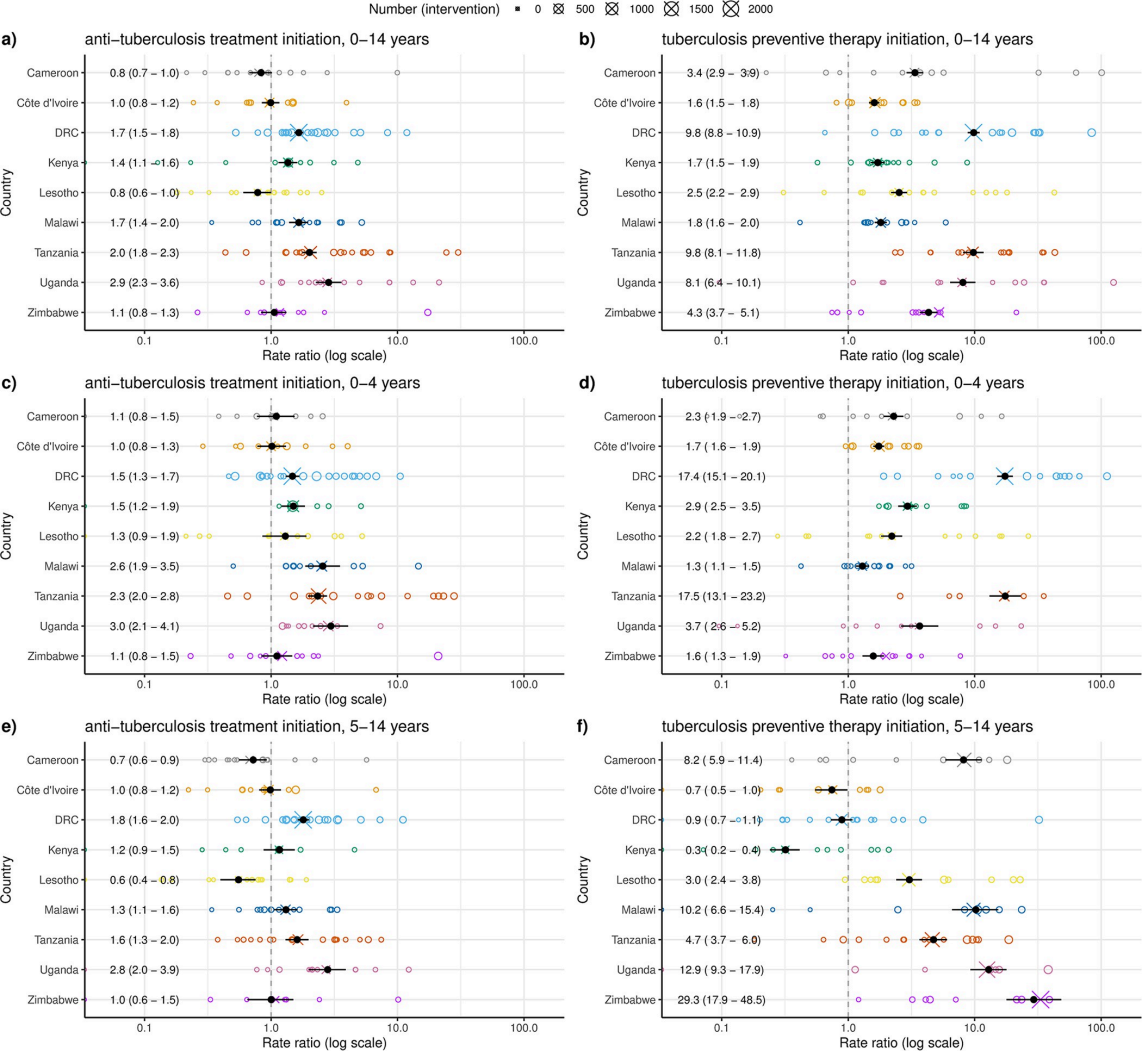
Estimates of the CaP-TB intervention effects on ATT and TPT initiation. Open circles are individual clinic empirical rate estimates, with size proportional to counts under intervention. Crosses are country-level empirical rate estimates based on aggregated counts and observation-time, with size proportional to counts under intervention. Black points and error bars are the country-level and meta-analytic estimates from the statistical model (median and 95% quantiles of posterior, also appearing as text on the left). For this Bayesian analysis, we do not present *p*-values. ATT, antituberculosis treatment; CaP-TB, Catalyzing Pediatric TB Innovations; TPT, tuberculosis preventive therapy.

Rate ratios for ATT and TPT initiation from the statistical model varied between 0.3 and 29.3, with larger increases typical for TPT than ATT, and larger ATT increases typical for ages 0 to 4 years (Fig 2). Inference diagnostics are presented in Table A2 in the S1 Appendix.

### Costs and cascades

The cascade of activities and overall cost associated with ATT and TPT initiation under SoC or during the intervention are shown in Table 1.

**Table 1:** Cascade of care results for each child initiating ATT or TPT under the CaP-TB package of interventions in comparison to SoC per country.

		Cameroon	Côte d’Ivoire	DRC	Kenya	Lesotho	Malawi	Tanzania	Uganda	Zimbabwe
ATT under SoC	Screened	248.08	175.77	31.84	253.14	389.8	388.55	136.23	213.24	168.71
	Presumptive TB	4.89	2.27	0.97	3.46	4.1	3.17	1.97	5.89	2.82
	Tested with Xpert	3.34	1.44	0.42	1.58	2.45	0.85	0.67	4.19	1.21
	TB diagnosed	1.01	1.05	1.03	1.01	1.01	1.03	1	1.01	1.01
	TB treated	1	1	1	1	1	1	1	1	1
	Cost per ATT initiation, $ (SD)	1,483 (532)	755 (263)	137 (41)	1,202 (437)	1,216 (424)	541 (174)	483 (161)	622 (181)	725 (250)
ATT under intervention	Screened	512.8	363.32	65.82	431.84	781.6	817.98	281.6	244.38	569.05
	Presumptive TB	10.11	4.69	2.01	5.9	8.21	6.67	4.08	6.75	9.52
	Tested with Xpert	9.47	4.07	1.18	3.43	7.09	1.38	1.9	4.09	7.9
	TB diagnosed	1.01	1.05	1.03	1.01	1.01	1.03	1	1.01	1.01
	TB treated	1	1	1	1	1	1	1	1	1
	Cost per ATT initiation, $ (SD)	4,849 (1,099)	2,941 (539)	528 (55)	3,116 (744)	10,923 (849)	1,959 (361)	1,852 (325)	1,786 (204)	4,835 (836)
TPT	Baseline ratio TPT:ATT initiations	1.20	2.43	0.45	1.67	1.90	2.35	0.41	0.90	1.02
	Intervention ratio TPT:ATT initiations	5.07	4.06	2.68	2.10	6.14	2.59	1.93	2.59	4.58
	TPT via HIV clinic*	37.31	0.00	4.66	11.60	15.88	29.21	36.13	45.71	42.11
	HHCM community based*	0.20	86.36	89.60	55.94	78.30	37.07	91.80	99.57	4.08
	Households screened	1.00	1.00	1.00	1.00	1.00	1.00	1.00	1.00	1.00
	Children screened	0.96	1.14	0.94	0.84	1.14	1.19	0.66	2.08	1.35
	Presumptive TB	2.35	2.14	2.66	2.07	1.98	1.78	3.71	4.13	1.99
	Diagnosed TB	0.12	0.14	0.11	0.10	0.14	0.15	0.08	0.26	0.17
	Started on TPT	0.03	0.04	0.03	0.03	0.04	0.04	0.02	0.07	0.05
	Cost per TPT initiation, US$ (SD)	44 (16)	39 (13)	63 (31)	45 (16)	33 (11)	20 (7)	71 (39)	39 (14)	57 (18)

Cost per ATT initiation and cost per TPT initiation were calculated by multiplying resource use for each cascade step by their respective unit costs and summing over the entire cascade. Unless specified, all values are counts (n) presented for one child starting ATT or TPT.

*Data are presented as percentages (%).

All costs are presented in 2020 United States dollars (US$).

ATT, antituberculosis treatment; CaP-TB, Catalyzing Pediatric TB Innovations; DRC, Democratic Republic of the Congo; SD, standard deviation; SoC, standard of care; TB, tuberculosis; TPT, tuberculosis preventive therapy.

Under SoC, the number of children screened per ATT initiation ranged between 32 in the DRC up to 390 in Lesotho. Between 1 and 6 of these children were considered presumptive for every child started on ATT. On average, between 0.4 and 4.2, Xpert tests were performed for every ATT initiation. Pretreatment loss to follow-up was below 5% in all settings. The associated activities implied that identification and treatment of 1 child under SoC had an economic cost ranging from US$137 in the DRC and US$1,483 in Cameroon.

Under the intervention, the number of children screened per ATT initiation increased to between 66 in the DRC and 818 in Malawi, the number of children identified with presumptive tuberculosis per ATT initiation to between 2 in the DRC and 10 in Cameroon, and Xpert testing to between 1.2 in the DRC and 9.5 in Cameroon per child initiated on ATT. Pretreatment loss to follow-up remained low. The increase in activities per treatment initiation and increase in activity unit costs under the intervention implied the cost per child initiating ATT under the intervention varied between US$528 in the DRC and US$10,923 in Lesotho.

The balance between ATT- and TPT-associated activities changed from between 0.5 and 2.4 TPT initiations per ATT initiation under SoC, to between 1.9 and 6.1 TPT initiations per ATT initiation under the combined intervention package (Table 1). The proportion of TPT initiations occurring in the HIV clinic under the intervention varied from 0% in Côte d’Ivoire to 46% in Uganda. The proportion of HHCM that was community-based, as opposed to facility-based, varied from 0.2% in Cameroon to 99.6% in Uganda. For every child initiating TPT under the intervention, between 0.7 and 2.1 households, and between 1.8 and 4.1 children were screened. For every 100 children initiating TPT under the intervention, between 8% and 26% across countries were considered to have presumptive tuberculosis, and between 2% and 7% were diagnosed with tuberculosis disease. The associated costs of these activities implied that under the intervention, the economic cost per child started on TPT varied between US$20 and US$71.

Unit costs for all activities are presented in Table A4 in S1 Appendix.

### Cost-effectiveness

Table 2 reports the outcomes, costs, and their changes going from SoC to intervention, for every 100 children under SoC initiating treatment (either ATT, TPT, or either ATT or TPT for the combined intervention).

**Table 2:** Healthcare resource use, health outcomes, costs, and cost-effectiveness of the CaP-TB package of interventions in comparison to SoC (baseline).

Country	SoC (per 100 treated in SoC)		Intervention (per 100 treated in SoC)		Difference (per 100 treated in SoC)							ICER (US$ per DALY averted)
	Started treatment	Cost	Started treatment	Cost	Started TPT	Incident TB	Started ATT	TB deaths	DALYs averted	Discounted DALYs averted	Cost	
	**ICF intervention**											
	(ATT)		(ATT)									
Cameroon	100	148,870 (80,773 to 237,775)	100 (85 to 117)	482,855 (325,136 to 679,515)	0	0	0 (−15 to 17)	0 (−5 to 4)	3 (−267 to 318)	1 (−114 to 135)	333,985 (150,790 to 544,283)	309,784
Côte d’Ivoire	100	75,914 (42,575 to 119,575)	85 (69 to 105)	250,683 (174,818 to 345,642)	0	0	−15 (−31 to 5)	2 (−5 to 7)	−94 (−427 to 345)	−45 (−187 to 141)	174,768 (88,472 to 274,884)	−3,895
DRC	100	13,679 (8,496 to 20,221)	166 (153 to 180)	87,763 (73,820 to 103,300)	0	0	66 (53 to 80)	−16 (−21 to −11)	989 (723 to 1,310)	418 (307 to 552)	74,084 (58,749 to 90,442)	177
Kenya	100	120,139 (66,940 to 188,677)	137 (115 to 163)	427,472 (278,309 to 613,333)	0	0	37 (15 to 63)	−14 (−23 to −6)	938 (394 to 1,609)	379 (159 to 649)	307,333 (145,370 to 498,556)	811
Lesotho	100	121,135 (67,906 to 192,031)	79 (60 to 104)	864,992 (639,347 to 1,152,211)	0	0	−21 (−40 to 4)	2 (−8 to 9)	−84 (−503 to 499)	−46 (−225 to 203)	743,856 (506,852 to 1,035,631)	−16,341
Malawi	100	54,133 (31,397 to 83,808)	167 (138 to 202)	326,948 (232,400 to 443,181)	0	0	67 (38 to 102)	−24 (−43 to −13)	1,676 (871 to 2,935)	677 (353 to 1,185)	272,815 (173,755 to 392,626)	403
Tanzania	100	48,371 (28,567 to 73,171)	202 (176 to 231)	375,230 (276,119 to 494,484)	0	0	102 (76 to 131)	−37 (−53 to −25)	2,544 (1,709 to 3,621)	1,029 (693 to 1,464)	326,860 (225,367 to 448,384)	318
Uganda	100	62,183 (39,829 to 90,053)	289 (228 to 364)	516,683 (384,847 to 682,803)	0	0	189 (128 to 264)	−62 (−98 to −37)	4,106 (2,479 to 6,524)	1,693 (1,028 to 2,679)	454,499 (320,360 to 621,552)	268
Zimbabwe	100	72,473 (40,309 to 113,551)	108 (85 to 136)	522,503 (362,148 to 732,851)	0	0	8 (−15 to 36)	−3 (−15 to 5)	219 (−345 to 966)	91 (−145 to 402)	450,030 (283,335 to 661,235)	4,968
	**HHCM intervention and HIV clinic preventive therapy intervention**											
	(TPT)		(TPT)									
Cameroon	100	4,372 (1,939 to 8,100)	141 (128 to 155)	14,358 (10,743 to 19,876)	41 (28 to 55)	−3 (−6 to −1)	0 (0 to 1)	−2 (−3 to −1)	100 (43 to 192)	41 (18 to 79)	9,986 (8,419 to 12,135)	242
Côte d’Ivoire	100	3,897 (1,831 to 6,868)	267 (227 to 311)	41,187 (33,359 to 51,223)	167 (127 to 211)	−11 (−20 to −5)	7 (4 to 10)	−6 (−11 to −4)	386 (214 to 678)	164 (91 to 287)	37,290 (30,614 to 45,538)	227
DRC	100	6,261 (2,261 to 14,130)	1,674 (1,453 to 1,933)	133,932 (106,926 to 171,609)	1,574 (1,353 to 1,833)	−109 (−211 to −38)	−11 (−66 to 23)	−47 (−91 to −20)	2,984 (1,273 to 5,829)	1,232 (525 to 2,407)	127,671 (101,596 to 163,660)	104
Kenya	100	4,549 (2,085 to 8,231)	269 (227 to 317)	26,469 (19,565 to 36,444)	169 (127 to 217)	−12 (−23 to −4)	−4 (−14 to 2)	−3 (−8 to −1)	242 (65 to 569)	98 (26 to 230)	21,920 (16,671 to 29,325)	224
Lesotho	100	3,261 (1,524 to 5,763)	266 (226 to 313)	20,745 (15,511 to 28,103)	166 (126 to 213)	−8 (−14 to −3)	6 (4 to 8)	−4 (−6 to −3)	243 (151 to 384)	106 (66 to 168)	17,484 (13,570 to 22,877)	164
Malawi	100	2,029 (901 to 3,689)	321 (244 to 433)	18,615 (14,193 to 25,430)	221 (144 to 333)	−3 (−5 to −1)	4 (2 to 7)	−3 (−4 to −1)	176 (93 to 299)	72 (38 to 123)	16,586 (12,980 to 22,189)	229
Tanzania	100	7,079 (2,243 to 17,152)	1,438 (1,108 to 1,873)	74,702 (46,843 to 118,484)	1,338 (1,008 to 1,773)	−72 (−144 to −25)	−26 (−95 to 16)	−23 (−58 to −4)	1,589 (275 to 4,008)	642 (113 to 1,617)	67,624 (40,455 to 109,539)	105
Uganda	100	3,943 (1,839 to 7,127)	735 (574 to 948)	144,924 (109,790 to 191,920)	635 (474 to 848)	−11 (−23 to −4)	16 (2 to 30)	−11 (−20 to −5)	741 (346 to 1,297)	308 (145 to 537)	140,981 (106,632 to 187,018)	457
Zimbabwe	100	5,684 (2,816 to 9,943)	1,207 (774 to 1,929)	154,235 (103,515 to 235,778)	1,107 (674 to 1,829)	−5 (−11 to −2)	17 (7 to 32)	−9 (−16 to −4)	541 (272 to 997)	235 (118 to 434)	148,551 (99,262 to 228,133)	632
	**Combined CaP-TB intervention package**											
	(ATT or TPT)*		(ATT or TPT)*									
Cameroon	100	69,905 (39,022 to 110,408)	122 (112 to 133)	226,831 (155,173 to 316,460)	22 (15 to 30)	−2 (−3 to −1)	0 (−7 to 8)	−1 (−3 to 1)	56 (−73 to 210)	23 (−32 to 88)	156,926 (73,737 to 252,201)	6,804
Côte d’Ivoire	100	24,909 (15,025 to 37,720)	214 (185 to 245)	102,311 (79,024 to 130,388)	118 (90 to 149)	−7 (−14 to −3)	1 (−5 to 7)	−4 (−8 to −1)	246 (82 to 488)	103 (33 to 206)	77,401 (51,793 to 107,234)	749
DRC	100	11,366 (7,437 to 16,262)	636 (567 to 717)	102,158 (88,785 to 117,820)	491 (422 to 572)	−34 (−66 to −12)	42 (23 to 57)	−25 (−40 to −16)	1,611 (1,022 to 2,546)	672 (428 to 1,058)	90,792 (77,239 to 106,498)	135
Kenya	100	47,808 (27,798 to 73,773)	219 (192 to 252)	176,543 (119,844 to 245,975)	106 (80 to 136)	−7 (−14 to −2)	11 (1 to 22)	−7 (−12 to −4)	503 (254 to 828)	203 (103 to 334)	128,735 (67,967 to 200,159)	634
Lesotho	100	43,847 (25,389 to 68,208)	202 (175 to 233)	311,429 (233,815 to 410,382)	109 (82 to 139)	−5 (−9 to −2)	−3 (−10 to 5)	−2 (−6 to 1)	131 (−31 to 355)	54 (−16 to 150)	267,582 (185,763 to 367,826)	4,949
Malawi	100	17,564 (10,679 to 26,435)	275 (220 to 355)	110,544 (81,983 to 145,911)	155 (101 to 234)	−2 (−3 to −1)	23 (14 to 34)	−9 (−15 to −5)	623 (366 to 1,020)	253 (149 to 413)	92,980 (63,185 to 128,913)	368
Tanzania	100	36,396 (22,220 to 54,374)	561 (461 to 687)	288,077 (217,445 to 372,763)	388 (292 to 514)	−21 (−42 to −7)	65 (38 to 90)	−33 (−49 to −22)	2,267 (1,504 to 3,361)	917 (610 to 1,355)	251,681 (178,952 to 338,223)	275
Uganda	100	34,519 (22,708 to 49,099)	501 (416 to 608)	340,097 (268,233 to 429,309)	302 (225 to 403)	−5 (−11 to −2)	107 (74 to 147)	−38 (−58 to −24)	2,508 (1,597 to 3,855)	1,035 (662 to 1,584)	305,578 (233,322 to 395,525)	295
Zimbabwe	100	38,697 (22,666 to 58,870)	664 (445 to 1,028)	336,265 (248,699 to 445,566)	560 (341 to 925)	−3 (−5 to −1)	12 (−1 to 28)	−6 (−13 to −1)	381 (58 to 816)	164 (28 to 347)	297,568 (208,997 to 407,694)	1,819

**Fig 3:**
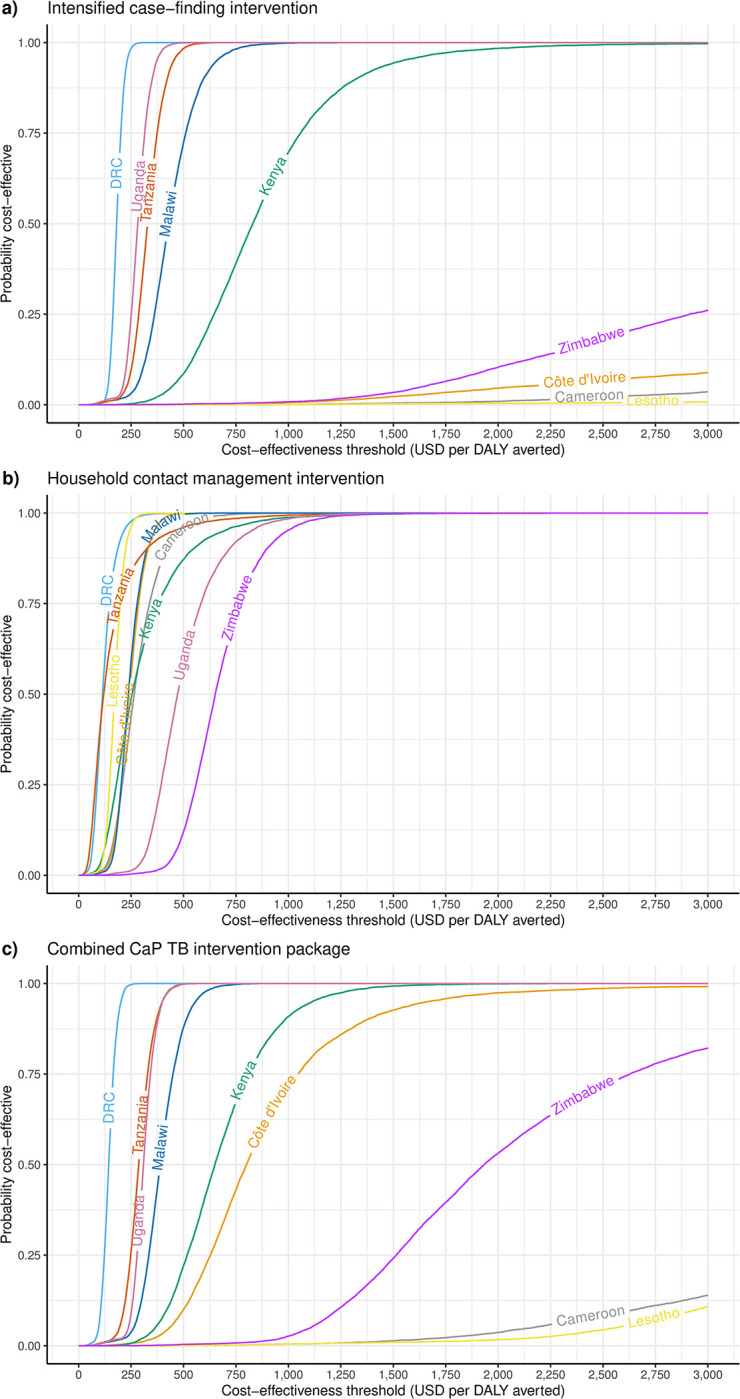
Cost-effectiveness acceptability curves for the CaP-TB package of interventions in comparison to SoC. The figure shows the probability that an intervention is cost-effective in each country, based on the proportion of simulations in which the comparison of the intervention to the SoC falls below the cost-effectiveness threshold shown on the horizontal axis. CaP-TB, Catalyzing Pediatric TB Innovations; DALY, disability-adjusted life year; DRC, Democratic Republic of the Congo; HIV, human immunodeficiency virus; SoC, standard of care; USD, United States dollar.

All outcomes are presented per 100 children initiating ATT or TPT at baseline. Data are presented as mean (95% uncertainty interval) unless otherwise stated. Uncertainty intervals are 95% percentiles from probabilistic sensitivity analysis, so we do not present *p*-values. See Fig 3 for uncertainty in cost-effectiveness. All costs are presented in 2020 United States dollars (US$).

ATT, antituberculosis treatment; CaP-TB, Catalyzing Pediatric TB Innovations; DALY, disability-adjusted life year; DRC, Democratic Republic of the Congo; HHCM, household contact management; HIV, human immunodeficiency virus; ICER, incremental cost-effectiveness ratio; ICF, intensified case finding; SoC, standard of care, TB, tuberculosis; TPT, tuberculosis preventive therapy.

*ATT or TPT comprises a mixture of each in the ratios presented in Table 1.

Across countries, for every 100 children starting ATT under SoC, a mean of between 79 and 289 started ATT under the intervention. This led to changes in the mean number of child tuberculosis deaths from between 2 more to 62 fewer for every 100 children starting ATT under SoC, corresponding to changes of between −46 and 1,693 discounted DALYs averted.

Across countries, for every 100 children starting TPT under SoC, a mean of between 141 and 1,674 started TPT under the intervention, leading to mean reductions of between 3 and 109 in child tuberculosis incidence. Changes in incidence together with additional children found through HHCM led to mean changes of between −26 and +17 children starting ATT per 100 TPT initiations under SoC. Per 100 TPT initiations under SoC across countries, the mean net deaths decreased by between 2 and 47, corresponding to mean gains of between 41 and 1,232 discounted DALYs averted.

Across countries, for every 100 children starting either ATT or TPT under SoC changes in the mean number of TPT initiations changed by between 22 and 560 and mean ATT initiations by between −3 to 107. These implied reductions in mean child tuberculosis deaths of between 1 and 38, corresponding to between 23 and 1,035 discounted DALYs averted.

Incremental costs combining changes in activities with changes in unit costs under the intervention, together with discounted changes in health, implied ICERs of between US$177 in DRC and US$309,784 in Cameroon per DALY averted for ICF interventions, where effective (Lesotho and Côte d’Ivoire treated fewer children during the intervention). For HHCM, ICERs ranged between US$104 in the DRC and US$632 in Zimbabwe per DALY averted. For the combined intervention package, ICERs ranged between US$135 in the DRC and US$6,804 in Cameroon per DALY averted (Table 2). Fig 3 shows the probability the interventions (ICF, HHCM, and the combined package) in each country are cost-effective for varying decision-maker thresholds. Threshold values where interventions first exceed 50% probability of being cost-effective are reported in Table A11 in S1 Appendix.

### Sensitivity analyses

Discounting DALYs averted by 5% increased ICERs by 50%, whereas a 0% discount rate decreased ICERs by 60%. Considering the reported changes in ATT/TPT success/completion between SoC and intervention as representative reduced ICERs by small amounts in countries with high baselines, but reduced the ICERs in Cameroon and Zimbabwe to US$1,351/DALY and US$823/DALY averted respectively. Neglecting the contribution of morbidity to DALYs changed ICERs by a median of 0.7% across countries (Table A10 in S1 Appendix).

## Discussion

In most countries, we found evidence of improvements in both ATT and TPT initiation and at the same time, higher rates of ATT success and TPT completion under the intervention. Our modelling suggested that improvements in treatment initiation (excluding completion improvement) implied the mean number of lives saved under the CaP-TB intervention package per 100 children initiating either ATT or TPT under SoC ranged from 1 to 38, with a median ICER relative to SoC of US$634/DALY averted, ranging from US$135 to US$6,804/DALY averted. Improvements in TPT initiation, from a low baseline, were relatively larger than the improvements in ATT initiation. ATT initiation improvements were greater in children aged 0 to 4 years, and we found the interventions were more cost-effective in children under 5, reflecting their higher risks of progressing to disease, going undiagnosed, and dying [1].

We found the HHCM intervention and HIV clinic TPT component of CaP-TB was typically more cost-effective than the ICF component and substantially improved the cost-effectiveness of the overall intervention in a number of countries. HHCM includes case finding as well as TPT, and this has been shown to be an important intervention component in achieving health impact [17]. CaP-TB, in common with other studies [20,21], found high tuberculosis disease prevalence among child household contacts, which accounts for the increases in ATT initiation for the TPT component in some countries despite reductions in incidence. Some recent studies of HHCM have found lower prevalence of tuberculosis among child household contacts [22], suggesting household tuberculosis coprevalence as an important factor to monitor since it may vary by setting.

Importantly, TPT and especially ATT initiation did not increase for all age groups in all clinics, and some country point estimates indicated decreases. The variation in intervention performance was a key driver of the variation in ICERs. Underlying reasons for worse performance will vary but include economic and operational issues as well as the influence of the Coronavirus Disease 2019 (COVID-19) pandemic. The heavy reliance on bacteriological diagnosis by healthcare workers in Cameroon and Côte d’Ivoire may have resulted in missed diagnoses due to challenges in obtaining samples, especially among young children, and the associated low sensitivity of currently available diagnostic tests. In Zimbabwe, economic crises and recurrent widespread healthcare worker strikes adversely impacted the intervention. Some variability may also reflect sampling noise due to low baseline numbers, especially for TPT in older age groups. National lockdowns in response to COVID-19 disrupted health services and attendance in many countries, and, together with increased stigma around respiratory symptoms and fear of infection, led to decreases in tuberculosis notifications, which were larger for children [23]. Although these disruptions led to a decrease in overall impact across all project countries, there was a slower recovery post-COVID-19 in some countries including Lesotho and Zimbabwe. Changes in response to COVID-19 may also have inflated the costs in some settings due to increased spending on personal protective equipment during the intervention.

The main limitation of our analysis is that our measure of effect is based on pre/post comparisons, rather than data from a randomised study, leaving it vulnerable to trends or shocks and lacking true causal attribution. Two cluster-randomised trials undertaken within the CaP-TB project have now completed recruitment. The INPUT study (NCT03862261) is a stepped-wedge trial in Cameroon and Kenya, evaluating the ICF component [24]. The CONTACT study (NCT03832023) compared 2 models of care for delivery of HHCM, with clusters in Cameroon and Uganda [25]. These studies should provide important randomised evidence on impact and cost-effectiveness that is less prone to confounding.

We were also not able to account for potential false-positive diagnoses, which might plausibly have increased under the ICF intervention. However, the proportion of tuberculosis diagnosed bacteriologically during the intervention increased compared with baseline. We also were not able to assess the separate cost-effectiveness of the household versus HIV clinic entry points for TPT. Understanding the relative impact and efficiency of these routes would be a valuable component of future work.

We made other simplifying assumptions in our modelling, such as assuming that all children with HIV were on ART, which is likely to be conservative for estimating health impacts because the reduction in mortality from ATT is smaller for children on ART. We used disease progression rates, relevant to TPT impact, which were based on a mixture of bacillus Calmette-Guérin (BCG) vaccination status [7]. Other modelling assumptions were also conservative: We only considered short-term risks of progression to tuberculosis disease and did not consider onward transmission from older children that would have generated additional indirect benefits, nor any household case finding in adults. We were able to explore the impact of a number of assumptions through sensitivity analyses described in the results.

Our costing is based on expenditure data from a real-world implementation, and our top-down unit cost estimates include investments in training and other aspects that are infrequently accounted for. Variations in cost-effectiveness therefore reflect differences in SoCs, heterogeneous real-world challenges, and include training and setup costs that are difficult to quantify in model-only analyses. Categorising costs by activity was not always straightforward, however, and for unallocated costs, an assumption of proportionality was used. For Cameroon, low denominators for some activities led us to use a bottom-up approach to estimate unit costs. We found substantial variation in the costs of initiating children on ATT or TPT, reflecting country differences in levels of CaP-TB activity implementation/support, prices, and cost allocation, but also country differences in numbers needed to screen. For ATT, clinician time spent symptom screening typically accounted for the majority of intervention costs, despite the low unit costs. We focussed on including unit cost uncertainty for the SoC in our analyses, as intervention unit costs were more precisely known.

Despite increases in both unit costs and intensity of child tuberculosis activities, the overall package of interventions had comparable cost-effectiveness to estimates for related interventions. The systematic review of tuberculosis screening by Empringham and colleagues [26] found ICERs of between US$281 and US$698 per DALY averted among the general population, US$619/QALY gained among children, and US$372 to US$3,718/DALY averted among close contacts. Economic evaluations specific to child tuberculosis have so far not evaluated costs and effects from real-world interventions, relying on model projections only. Analyses of use of additional diagnostic tools have typically found ICERs in the region of $100/DALY averted [12–14]. Analyses of TPT-based interventions have found ICERs in between US$237 and US$538/DALY averted for different screening strategies at clinic [15], between US$100 and US$1,300/DALY averted across 12 countries for short-course TPT in child household contacts [16], and a global mean between US$703 and US$1,208/DALY averted for different strategies for household contacts of rifampicin-resistant tuberculosis [17]. Ochalek and others have indirectly estimated the marginal cost-effectiveness of interventions currently adopted by health systems for large numbers of countries across all types of intervention [27,28]. Ultimately, the choice of any cost-effectiveness threshold is that of the decision-maker, and cost-effectiveness acceptability curves such as those in Fig 3 can be used to assess the probability of cost-effectiveness at any threshold.

Such evidence is key to meeting the priority of Roadmap towards Ending Tuberculosis in Children and Adolescents to establish decentralised and family-centred, integrated models of care [29]. Despite a wealth of practical experience documented in the associated operational handbook [11], the 2022 WHO guidelines on management of tuberculosis in children and adolescents included only a conditional recommendation on decentralised delivery, due to a lack of evidence [11]. It is important that efforts to improve tuberculosis care for children and adolescents are documented and reported in order to expand the evidence base and share experience.

To our knowledge, our analysis is the first cost-effectiveness analysis of interventions for paediatric tuberculosis that makes use of empirical estimates of intervention impact and costs, in this case interventions that have been deployed on a large scale across multiple countries. We found a median ICER for the CaP-TB intervention package across countries US$634 per DALY averted, but the large variability between countries highlights the importance of local context in determining impact and cost-effectiveness.

## Supporting information

S1 CHEERS Checklist Reporting guidance for health economic evaluations. (DOCX)

S1 Appendix Supplementary methods and results. (PDF)

## Abbreviations

ATT: antituberculosis treatment
BCG: bacillus Calmette-Guérin
CDR: case detection ratio
CFR: case fatality ratio
CHW: community healthcare worker
COVID-19: Coronavirus Disease 2019
DALY: disability-adjusted life year
DRC: Democratic Republic of the Congo
HHCM: household contact management
ICER: incremental cost-effectiveness ratio
ICF: intensified case finding
SoC: standard of care
TPT: tuberculosis preventive therapy
UI: uncertainty interval
WHO: World Health Organization
