# Supplementary material for: Cost-effectiveness analysis of interventions to improve diagnosis and preventive therapy for paediatric tuberculosis in 9 sub-Saharan African countries: A modelling study
Source: PLoS Med. 2023 Sep 6;20(9):e1004285. doi: 10.1371/journal.pmed.1004285 (PMC10511115; doi:10.1371/journal.pmed.1004285)
Supplement: S1 Appendix — (PDF) [file pmed.1004285.s002.pdf]

Cost-effectiveness analysis of interventions to improve diagnosis and preventive therapy for paediatric tuberculosis in nine sub-Saharan African countries: a modelling study

Nyashadzaishe Mafirakureva[1], Sushant Mukherjee[2], Mikhael de Souza[2], Cassandra Kelly-Cirino[2], Mario J.P. Songane[2], Jennifer Cohn[3], Jean-François Lemaire[2], Martina Casenghi[2], Peter J. Dodd[1]

1. Sheffield Centre for Health and Related Research, University of Sheffield, UK
2. Elizabeth Glaser Pediatric AIDS Foundation
3. Division of Infectious Diseases, University of Pennsylvania School of Medicine, USA

Description of pre/post data used to quantify effect **2**
Analysis of intervention effects **10**
Statistical model specification 10
Inference diagnostics 11
Costing and care cascades **12**
Overview 12
Unit costs and care cascades 15
Modelled outcomes & health economic approach **21**
Overview 21
Reproducibility and pre-registration 22
Details of modelling approach 22
Supplementary results **26**
Sensitivity analyses **31**
References **37**

Description of pre/post data used to quantify effect

The cost-effectiveness analysis utilised evidence generated in 9 project countries in the African region. The following data from the 'TIPPI' monitoring and evaluation study within the CaP-TB study were used in this analysis. For sites with both baseline and prospective data available: monthly rates per site (including absolute numerator and denominator or observation time) of TB notifications, ATT initiations, and TPT initiations. At country aggregated-level: numbers of a) sites reporting, b) children screened for symptoms, c) presumptive TB identified, d) presumptive TB tested with Xpert, e) diagnosed with TB, f) treated for DS- TB, g) index cases with contact tracing done, h) TPT initiations among contacts, i) TPT initiation among HIV entry point, and j) TPT initiation all.

For TB diagnosis, the median/IQR site-time was 33.04 (30.51 - 34.29) with a total of 5256 months during baseline and 9010 months under intervention. Baseline site TB diagnosis rates ranged 0.27 - 2.26 at baseline and 0.26 - 3.55 under intervention. 71.92% of sites had higher rates of TB diagnosis for children aged 0-4 years; 56.16% of sites for children aged 5-14 years.

For ATT initiation, the median & IQR site-time was 33.04 (30.51 - 34.29) with a total of 5256 months during baseline and 9010 months under intervention. Baseline site ATT initiation rates ranged 0.27 - 2.08 at baseline and 0.26 - 3.45 under intervention. 70.55% of sites had higher rates of ATT initiation for children aged 0-4 years; 57.53% of sites had higher ATT initiation rates for children aged 5-14 years. ATT success rates at baseline (under SoC) and under intervention are shown in Table A1.

For TPT initiation, the median & IQR site-time was 33.01 (30.28 - 34.29) with a total of 5436 months during baseline and 9152 months under intervention. Baseline site TPT initiation rates ranged 0.24 - 3.34 at baseline and 1.62 - 9.25 under intervention. 88.08% of sites had higher rates of TPT initiation for children aged 0-4 years; 74.17% of sites had higher TPT initiation rates for children aged 5-14 years. TPT completion rates at baseline (under SoC) and under intervention are shown in Table A1.

Individual site pre/post data for ATT and TPT are shown in Figure A1 through to Figure A6.

Table A1 Average TB treatment success rates and TPT completion rates per country. ATT=anti-tuberculosis treatment, DRC=Democratic Republic of the Congo, SoC=standard of care, TB=tuberculosis, TPT=tuberculosis preventive therapy.

**country**	**ATT success, SoC (%)**	**ATT success, intervention (%)**	**PT completion, SoC (%)**	**PT completion, intervention (%)**
Cameroon	66.7	88.7	43.4	94.6
Côte d'Ivoire	87.9	91.7	68	85
DRC	86.9	94	88.5	91.3
Kenya	67.9	83.6	63.9	92.8

Lesotho	85.1	85.1	32.7	83.3
Malawi	80.8	89.1	93.4	98.9
Tanzania	63	94.7	37.5	99
Uganda	68.1	84.7	73.7	95.3
Zimbabwe	48.9	87.4	72.7	86.8

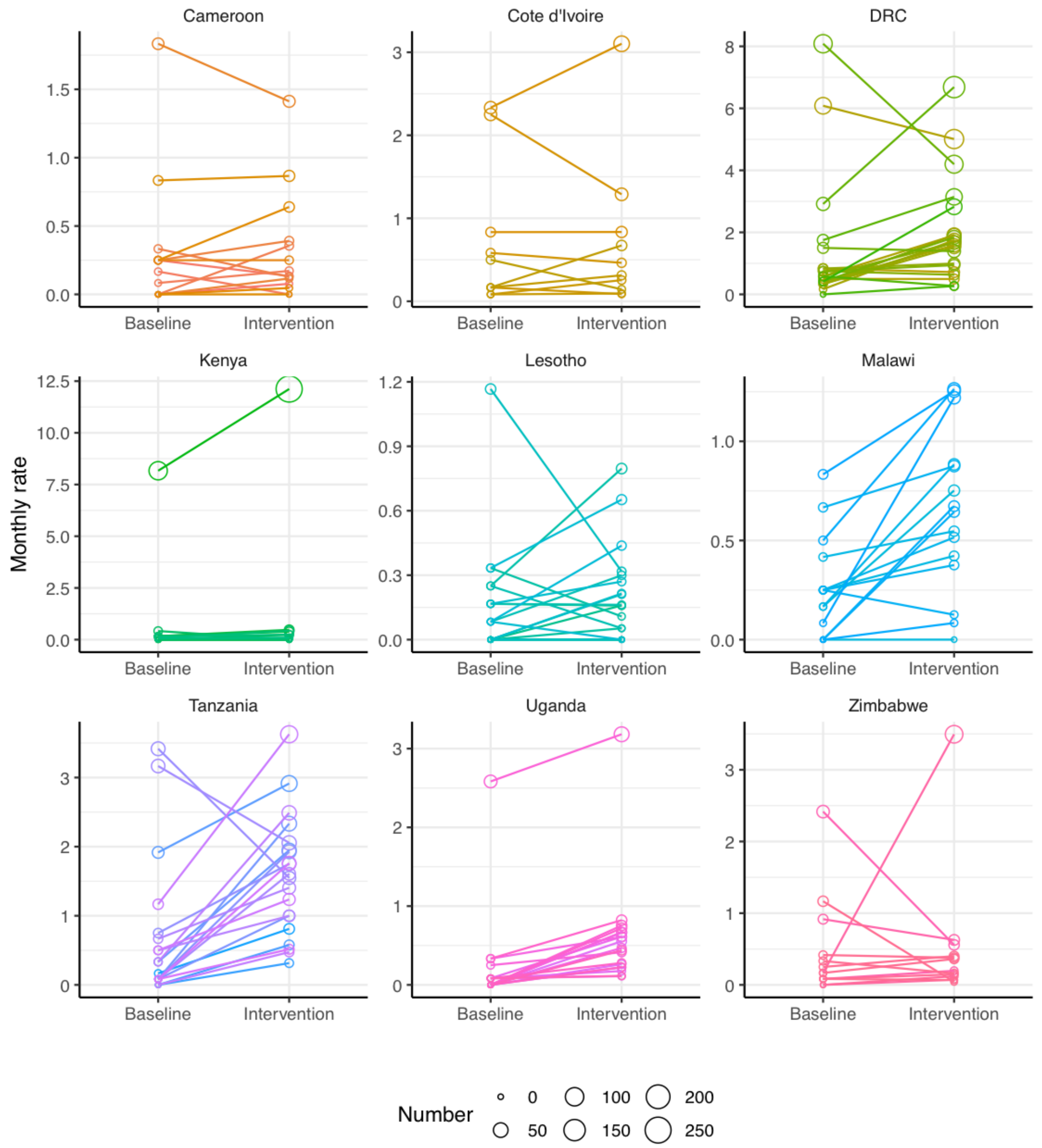

Figure A1 Estimates of the Cap TB intervention effects on TB treatment among children aged 0-4 years. Individual sites are joined with lines. CaP-TB=Catalyzing Pediatric TB Innovations, DRC=Democratic Republic of the Congo, TB=tuberculosis.

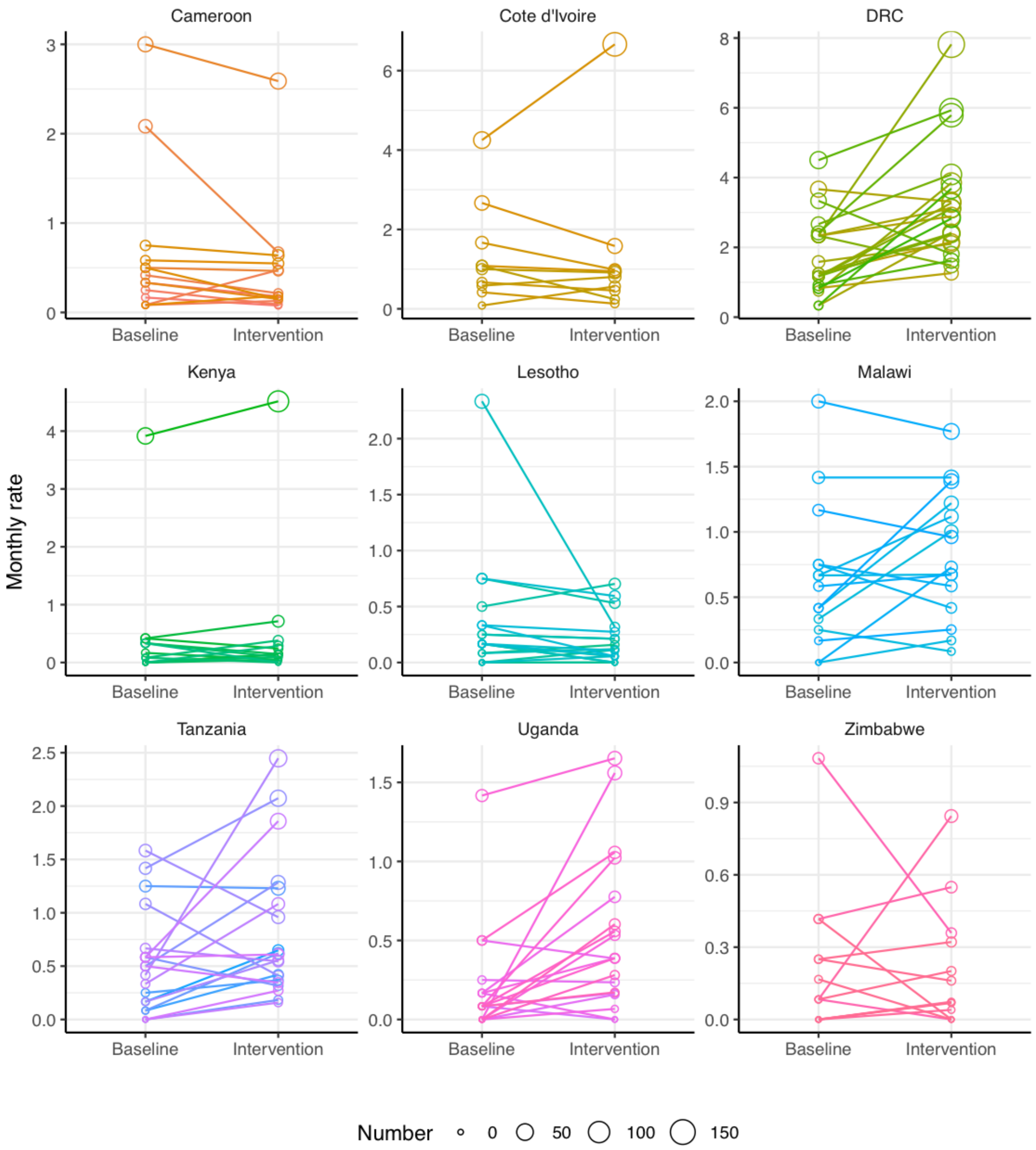

Figure A2 Estimates of the Cap TB intervention effects on TB treatment among children aged 5-14 years. Individual sites are joined with lines. CaP-TB=Catalyzing Pediatric TB Innovations, DRC=Democratic Republic of the Congo, TB=tuberculosis.

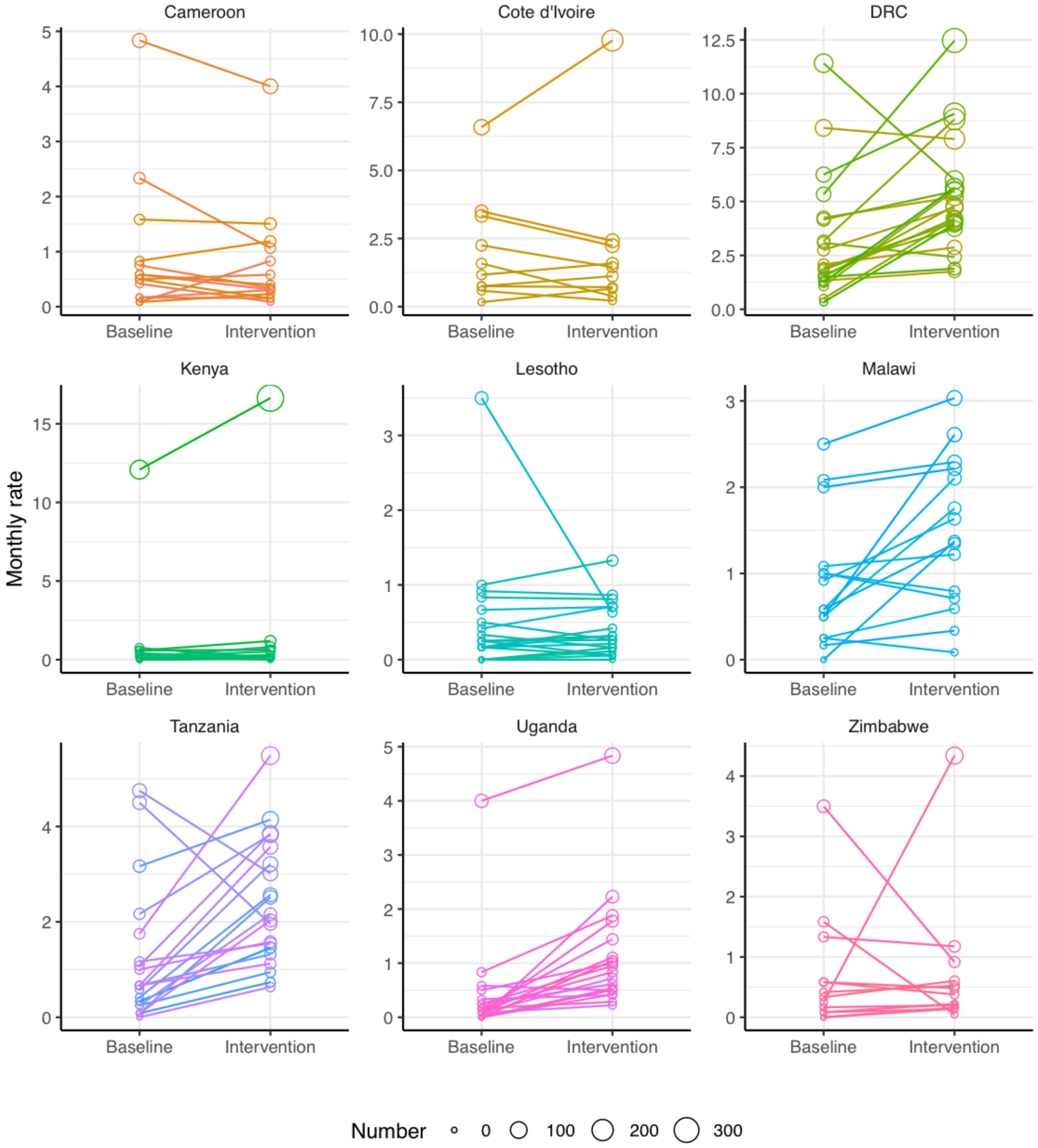

Figure A3 Estimates of the Cap TB intervention effects on TB treatment among children aged 0-14 years. Individual sites are joined with lines. CaP-TB=Catalyzing Pediatric TB Innovations, DRC=Democratic Republic of the Congo, TB=tuberculosis.

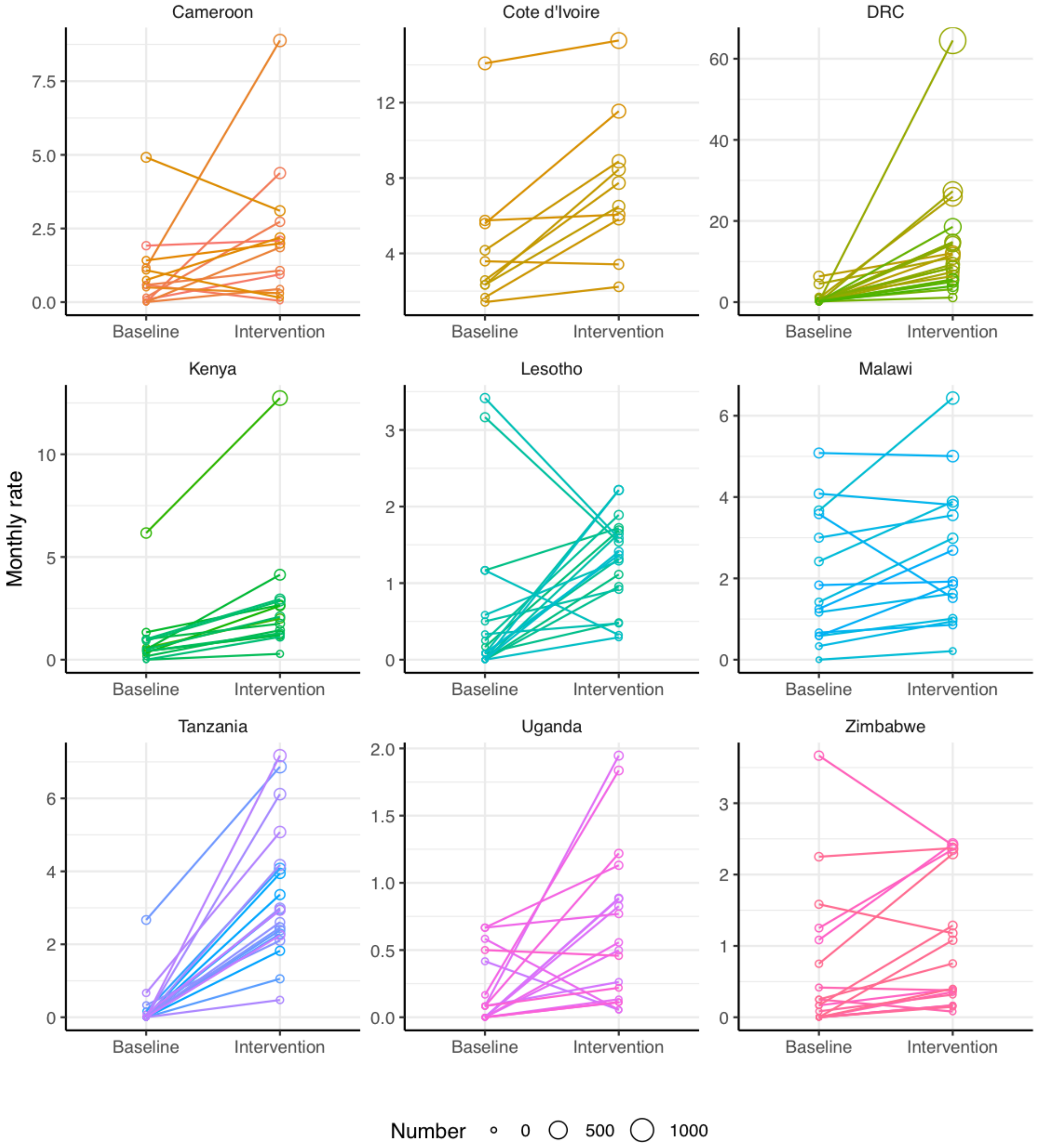

Figure A4 Estimates of the Cap TB intervention effects on TB preventive therapy among children aged 0-4 years. Individual sites are joined with lines. CaP-TB=Catalyzing Pediatric TB Innovations, DRC=Democratic Republic of the Congo, TB=tuberculosis.

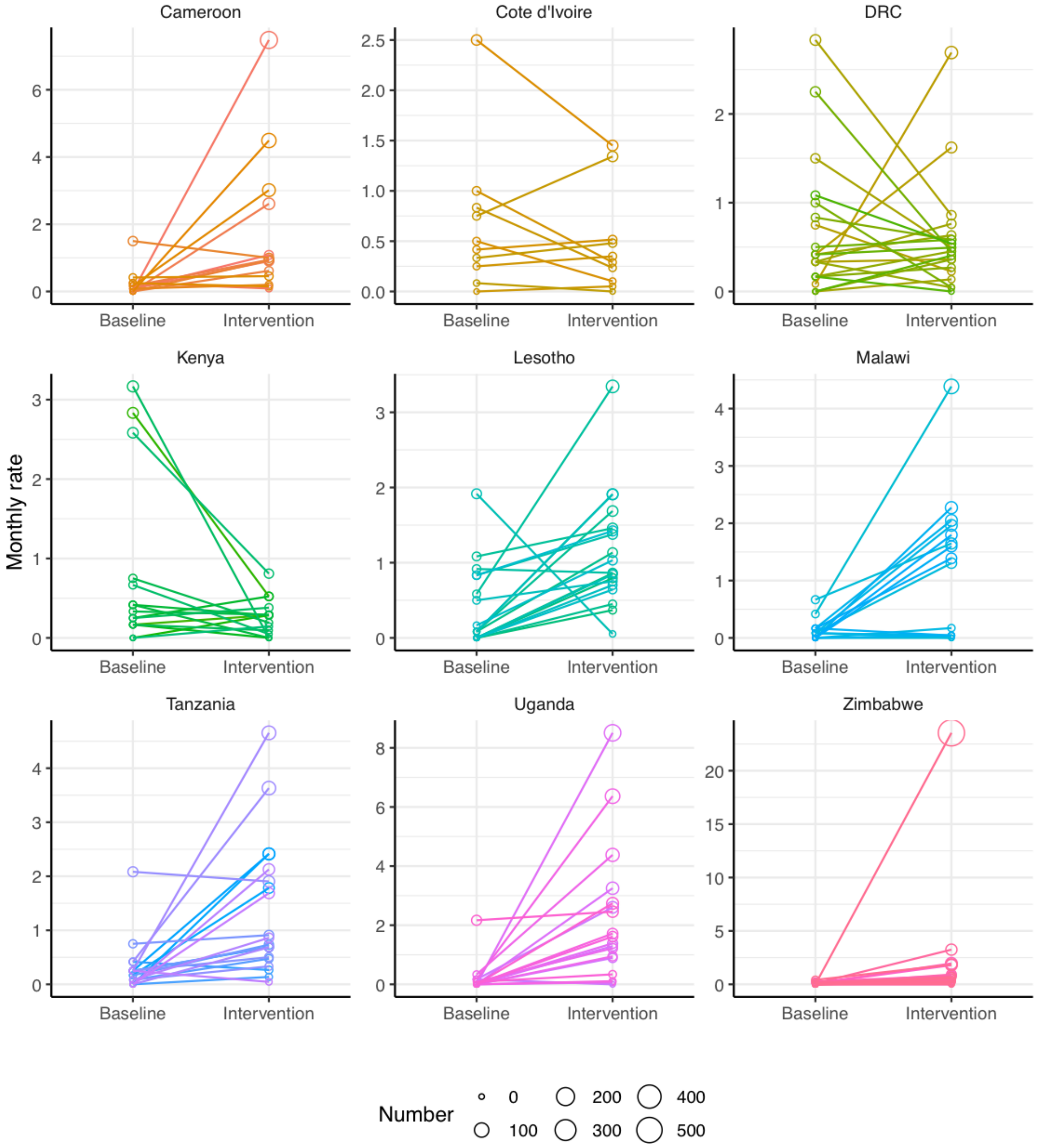

Figure A5 Estimates of the Cap TB intervention effects on TB preventive therapyamong children aged 5-14 years. Individual sites are joined with lines. CaP-TB=Catalyzing Pediatric TB Innovations, DRC=Democratic Republic of the Congo, TB=tuberculosis.

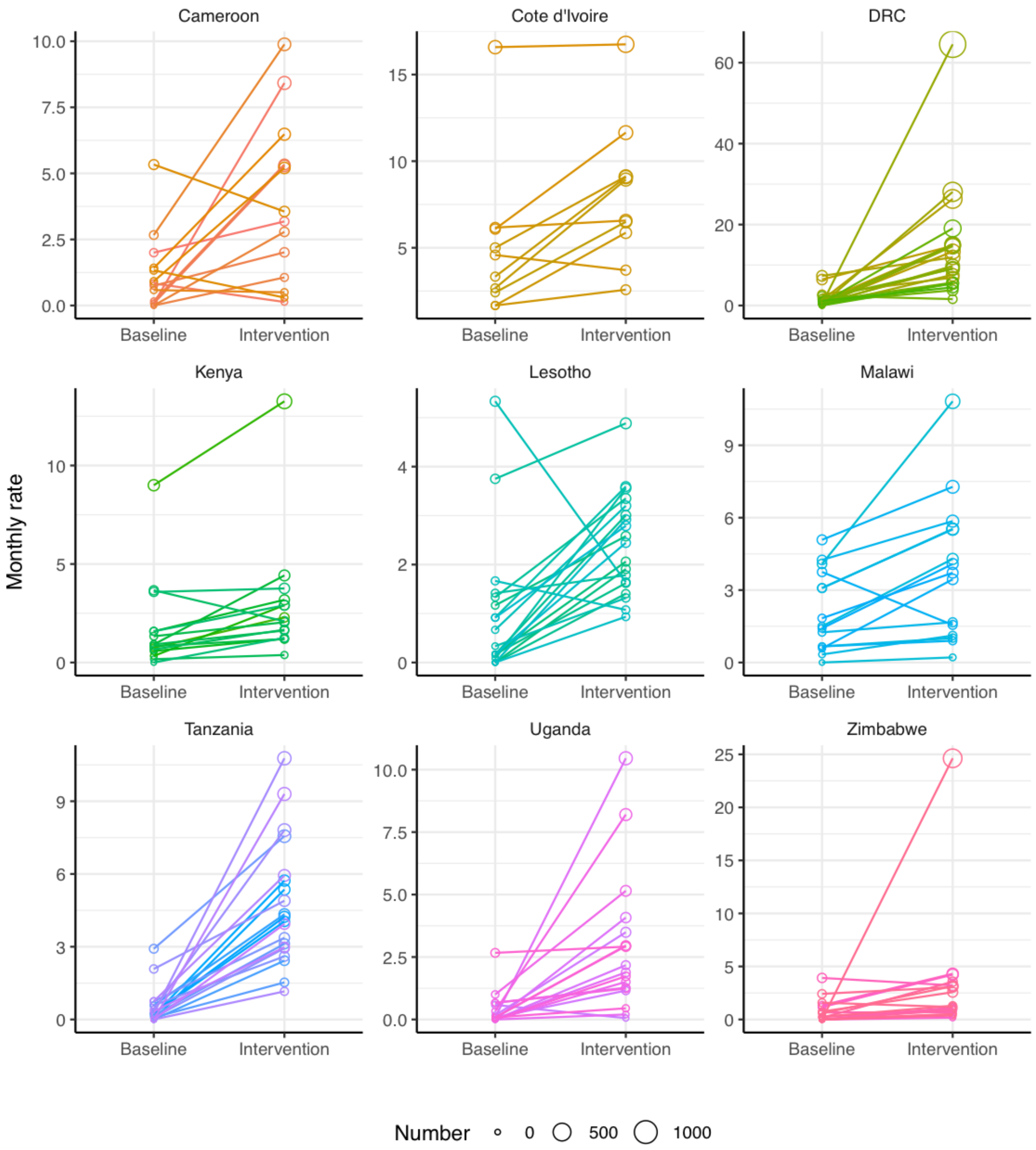

Figure A6 Estimates of the Cap TB intervention effects on TB preventive therapy among children aged 0-14 years. Individual sites are joined with lines. CaP-TB=Catalyzing Pediatric TB Innovations, DRC=Democratic Republic of the Congo, TB=tuberculosis.

Analysis of intervention effects

The effect of the intervention as an incidence rate ratio (IRR) was quantified by statistical analysis of before and after data at the site level. This focussed on changes in 2 quantities, stratified by age group (<5 years and 5-14 years): 1) the rate of initiating children on anti-TB treatment (ATT); 2) the rate of initiating children on TPT. These quantities and intervention impacts were modelled with a Bayesian generalised linear mixed-effects model, with checks against a country-wise random effects meta-analysis. For all quantities, the counts were modelled as Poisson distributed counts with means equal to the product of rates and observation-time.

Statistical model specification

We used a Bayesian generalised linear model with site-level random effects.

Let C_i be the event count for record $i \in \{1, ..., N\}$, and (in the notation of Gelman & Hill[1]) let $j[i]$ be the index for the country of the *i*-th record ($j \in \{1, ..., J\}$) and $k[i]$ be the site of the *i*-th record, and T_i be the corresponding observation-time.

We use the model

$$C_i \sim \text{Poisson}(\lambda_\text{i})$$

where

$$\log(\lambda_i) = \log(T_i) + \alpha_0 + \alpha_{j[i]} + \beta_{j[i]} \times 1(i \in \text{intervention}) + \varepsilon_{k[i]}$$

with priors

$$\alpha_0, \alpha_j, \beta_j \sim \text{Normal}(0, 2.5)$$
$$\varepsilon_k \sim \text{decov}(1, 1, 1, 1).$$

Country-level IRRs were computed as

$$IRR_j = \exp(\beta_j).$$

Inference was performed using rstanarm[2] using the default priors noted above, and 2 chains of length 6,000 (the first 1,000 iterations of each discarded as burn in). A posterior sample of 10,000 was used as the basis for modelling.

Inference diagnostics

Gelman-Rubin R-hat statistics and effective sample sizes (ESS) suggested good convergence (see Table A2).

Table A2 Effective sample size and Rhat convergence statistics across parameters for each model. ATT=anti-tuberculosis treatment, ESS=effective sample sizes, TPT=tuberculosis preventive therapy.

**quantity**	**age**	**median Rhat**	**min Rhat**	**max Rhat**	**median ESS**	**min ESS**	**max ESS**
TPT	0-14	1.000162	0.999815	1.001323	2948	1734	8178
TPT	0-4	1.000056	0.9998013	1.001433	3131	1798	10345
TPT	5-14	1.000848	0.999827	1.006726	1638	725	5271
ATT	0-14	1.000726	0.9998091	1.003561	2503	757	7377
ATT	0-4	1.000766	0.9998015	1.012881	1711	536	6980
ATT	5-14	1.000516	0.9998059	1.003557	2315	494	9067

Costing and care cascades

Overview

Costs for the standard of care for TB diagnosis, ATT and TPT were derived from published literature sources. Country-specific costs were used where possible otherwise costs were transferred from other countries by applying relevant purchasing power parity conversion factors. Table A4 shows the assumptions used in deriving unit costs for the standard of care. All historical costs were exchanged to US$ using the World Bank-based period average exchange rates at the time of the costing,[3] and then adjusted to 2020 prices using US inflation rates.[4]

We estimated incremental costs of the intervention by considering all CaP-TB-supported costs as incremental to the standard of care unit costs in the cascade of care for childhood TB management. We defined incremental costs in this context as the additional costs that a public health system would incur in order to improve utilisation (through increased access) of paediatric tuberculosis services, beyond what the system currently provides. We used an activity-based, top-down approach to costing, using budget and expenditure data, together with input from field teams on expenditure breakdowns to estimate intervention costs. Bottom-up estimates were calculated and applied for one activity in one country that was implemented late on during the intervention resulting in very low volumes and unreasonably high unit costs.

CaP TB budget data were organised in cost categories and sub-categories, excluding costs not directly relevant to project implementation, and then the fractional contribution of each subcategory to the overall cost category was calculated, assuming that this remained constant over time. These fractions were applied to actual expenditure data from EGPAF financial records to disaggregate expenditure to the sub-category level. However, for TB medicines and diagnostics, exact costs incurred by each country for each item purchased were taken from expenditure data directly. For both budget and expenditure data, EGPAF overhead costs, staff costs for time spent on donor report/award management (EGPAF related) and CaP TB project specific monitoring and evaluation (M & E) infrastructure costs were excluded because these were assumed not relevant to MoH-led M&E routine implementation. The tool used to categorise and allocate costs is shown in Table A3.

Costs were then assigned to individual activities across the full range of interventions using the following groups: project set-up and demand generation; community-based household contact tracing; facility-based household contact tracing; screening of non-contacts in HIV entry points; screening of non-contacts in non-HIV entry points; TB evaluation and diagnosis; TB treatment; TB preventive therapy; evaluation (including M & E and research to allow exclusion); and finally, program management. Country teams were asked to assess the proportion of costs for category and sub-category that was associated with each activity. Costs disaggregated across categories and activities were then used to develop unit costs for each direct patient care activity. All research related costs were identified and excluded from the final analysis in order to represent real world implementation. All costs were available in US$ and were adjusted to 2020 prices using US inflation rates.

Costs for intervention and standard of care were therefore calculated as sums over activities:

$$\text{Cost, SoC} = \sum(\text{activity, SoC}) \times (\text{SoC activity unit cost})$$

$$\text{Cost, intervention} = \sum(\text{activity, intervention}) \times (\text{SoC activity unit cost} + \text{incremental activity unit cost})$$

Table A3 Cost data allocation to TB activities with illustrative line cost items. Cost data allocation tool used to allocate Cap TB project expenditures to TB activities with illustrative line cost items. CXR=chest x-ray, MoH=ministry of health, M&E=monitoring and evaluation, TB=tuberculosis, TPT=tuberculosis preventive therapy.

	**Project setup (pre-implementa tion**		**Contact tracing and TB symptom screening**		**TB evaluation and diagnosis**					**TB treatment**	**TPT**	**Site Supervision**	**Evaluation**		**Program Management**
**Resource categories**	Project planning	Staff training	Community based	Facility based	Clinical evaluation	Sample collection	Sputum smear	Xpert	CXR	TB treatment	TPT	Site Supervision	M&E	Research	Program Management
**Human resources**															
Program staff															
MoH staff															
Ad-hoc staff															
**Travel**															
Contact Tracing - Transport															
Site support and supervision															
Travel for training & workshops															
M&E															
**Equipment and Supplies**															
Reagents and chemicals															
Consumables															
Medicines															
Other health products															
**Trainings and Meetings**															
On-Site Training															
Training of trainers															
Community health worker training															
Review meetings															
**Other Implementation Costs**															
CXR subsidies															
Sample transportation															
Consultants for Site Support															
Translation of Guidelines															
**Total activity costs**															

Unit costs and care cascades

The data in Table A4 show the unit costs for each country and activity under the standard of care, and the incremental unit costs for the intervention (ie the unit costs under intervention are the sum of standard of care unit costs and incremental unit costs). We were unable to identify an appropriate value for household contact tracing in the literature, and so considered this in sensitivity analysis.

Table A4 Assumptions used for estimating unit costs in the standard of care and estimated unit costs (USD, standard deviation in brackets) by country and activity. CMR=Cameroon, CDI=Côte d'Ivoire, DRC=Democratic Republic of the Congo, KEN=Kenya, LSO=Lesotho, MWI=Malawi, TZA=Tanzania, UGA=Uganda, USD=United States dollar, ZWE=Zimbabwe. DS-TB=drug sensitive tuberculosis, TB=tuberculosis, WHO-CHOICE= World Health Organization CHOosing Interventions that are Cost-Effective, RHZE= rifampicin, isoniazid, pyrazinamide and ethambutol, RH=rifampicin and isoniazid, SoC=Standard of Care, SD=Standard deviation, TPT=tuberculosis preventive therapy, TB= tuberculosis.

**Cost parameter**	**Description**	**Unit cost parameter estimation assumptions**	**Country**	**SoC mean unit cost (SD)**	**Intervention Incremental Cost**
Community-based household contact investigation	Cost of active TB contact tracing and screening in the household	Assumed mostly passive case finding at baseline hence no cost of active TB contact tracing and screening.	CMR	0.00 (-)	11.3
			CDI	0.00 (-)	62.12
			DRC	0.00 (-)	30.51
			KEN	0.00 (-)	94.56
			LSO	0.00 (-)	28.3
			MWI	0.00 (-)	111.91
			TZA	0.00 (-)	44.71
			UGA	0.00 (-)	231.56
			ZWE	0.00 (-)	399.61
Facility-based household contact investigation	Cost of active TB contact tracing and screening in the facility	Equivalent to the country-specific cost of one outpatient visits to a health centre with no beds based on WHO-CHOICE estimates inpatient and outpatient health service delivery.	CMR	5.03 (2.21)	42.38
			CDI	3.52 (1.51)	69.48
			DRC	1.54 (0.62)	2.16
			KEN	4.13 (1.79)	54.16
			LSO	2.72 (1.14)	9.3
			MWI	1.14 (0.45)	56.72
			TZA	2.73 (1.15)	28.48
			UGA	2.02 (0.83)	0
			ZWE	3.43 (1.47)	230.35

TB symptom screening in HIV clinic	The cost of TB symptom screening in children presenting to the HIV clinic		CMR	5.03 (2.21)	0.17
			CDI	3.52 (1.51)	0
			DRC	1.54 (0.62)	0.02
			KEN	4.13 (1.79)	3.17
			LSO	2.72 (1.14)	1.5
			MWI	1.14 (0.45)	0.13
			TZA	2.73 (1.15)	0
			UGA	2.02 (0.83)	0.01
			ZWE	3.43 (1.47)	0
TB symptom screening in non-HIV clinic	The cost of TB symptom screening in children presenting to non-HIV clinics		CMR	5.03 (2.21)	0.3
			CDI	3.52 (1.51)	0.22
			DRC	1.54 (0.62)	0.07
			KEN	4.13 (1.79)	1.2
			LSO	2.72 (1.14)	8.61
			MWI	1.14 (0.45)	0.62
			TZA	2.73 (1.15)	0.79
			UGA	2.02 (0.83)	0.96
			ZWE	3.43 (1.47)	0
Presumptive TB evaluation	The cost associated with clinical assessment for the diagnosis of TB. This can occur in children self-presenting or referred to the facility through contact tracing.		CMR	5.03 (2.21)	31.93
			CDI	3.52 (1.51)	38.02
			DRC	1.54 (0.62)	0.16
			KEN	4.13 (1.79)	0
			LSO	2.72 (1.14)	28.87
			MWI	1.14 (0.45)	9.26
			TZA	2.73 (1.15)	14.58
			UGA	2.02 (0.83)	30.29
			ZWE	3.43 (1.47)	53.1
Sample collection	The costs for collecting self-expectorated sputum for bacteriological testing.	Equivalent to cost of materials and staff time (based on data from CMR & KEN) required to collect a single self-expectorating sputum sample. Transferred to other countries using the purchasing power parity (PPP) adjustment factors.	CMR	1.36 (0.01)	56.7
			CDI	1.29 (0.01)	81.55
			DRC	1.65 (0.01)	79.96
			KEN	1.31 (0.01)	69.4
			LSO	1.29 (0.01)	138.91
			MWI	1.21 (0.01)	100.84
			TZA	1.31 (0.01)	209.44
			UGA	1.13 (0)	55.21
			ZWE	1.61 (0.01)	112.5
Chest X-ray	The cost of chest X-ray	Applied a mean chest x-ray cost from 2014 in Tanzania, Uganda and	CMR	7.35 (0.73)	3.02

		Zimbabwe and transferred to other countries using the PPP adjustment factors	CDI	6.97 (0.69)	10.53
			DRC	8.9 (0.88)	0.2
			KEN	7.06 (0.7)	0.33
			LSO	6.96 (0.69)	0.86
			MWI	6.54 (0.65)	0.15
			TZA	7.1 (0.71)	0.89
			UGA	6.11 (0.61)	0.14
			ZWE	8.69 (0.86)	0
Xpert testing	The cost for testing a single sample using the GeneXpert.	Applied 2014 GeneXpert test cost from Uganda and transferred to other countries using the PPP adjustment factors.	CMR	27.44 (13.37)	47.62
			CDI	26.01 (12.67)	40
			DRC	33.24 (16.2)	20.65
			KEN	26.36 (12.85)	0.33
			LSO	25.98 (12.66)	33.2
			MWI	24.41 (11.89)	14.34
			TZA	26.51 (12.92)	110.08
			UGA	22.82 (11.12)	22.71
			ZWE	32.44 (15.81)	12.03
TB treatment	The total cost treatment for a child diagnosed with DS-TB and initiated on treatment.	Comprises of anti-TB drugs estimated using weight band-based dosing and applying the Global Drug Facility unit costs assuming treatment duration of 6 months for DS-TB (2RHZE + 4RH) and 10 treatment follow-up visits valued at the country-specific cost of a single outpatient visit to a health centre with no beds. Ten treatment follow-up visits were obtained by assuming biweekly visits during the intensive phase, monthly visits during the continuation phase and 2 additional visits per child during TB treatment.	CMR	110.32 (52.11)	158.56
			CDI	95.25 (45.11)	130.42
			DRC	75.49 (36.26)	3.59
			KEN	101.41 (47.95)	13.66
			LSO	87.24 (41.46)	343.39
			MWI	71.52 (34.56)	13.3
			TZA	87.33 (41.5)	10.54
			UGA	80.29 (38.36)	62.76

			ZWE	94.37 (44.7)	518.36
TPT treatment	The total cost treatment for a child initiated on TPT.	Comprises of TPT drugs estimated using weight band-based dosing and applying the Global Drug Facility unit costs assuming treatment duration of 3 months (3RH) and 5 treatment follow-up visits valued at the country-specific cost of a single outpatient visit to a health centre with no beds. five treatment follow-up visits were obtained by assuming biweekly visits during the first month, monthly visits during the subsequent months and 1 additional visit per child during TPT.	CMR	34.43 (15.69)	27.73
			CDI	26.89 (12.19)	46.44
			DRC	17.01 (7.76)	35.33
			KEN	29.97 (13.61)	3.41
			LSO	22.89 (10.36)	25.08
			MWI	15.02 (6.91)	8.19
			TZA	22.93 (10.38)	4.3
			UGA	19.41 (8.81)	26.66
			ZWE	26.45 (11.99)	58.30

The cascade of care for ATT is shown in Table 1 in the main article. The number of children per child started on ATT that were screened, identified as presumptive TB, and tested with Xpert all rose under the intervention, as did the cost of all activities (including ATT) per child treated (see Figure A8). The bulk of the costs per child treated for TB, particularly under the intervention, were associated with screening activities (see Figure A7).

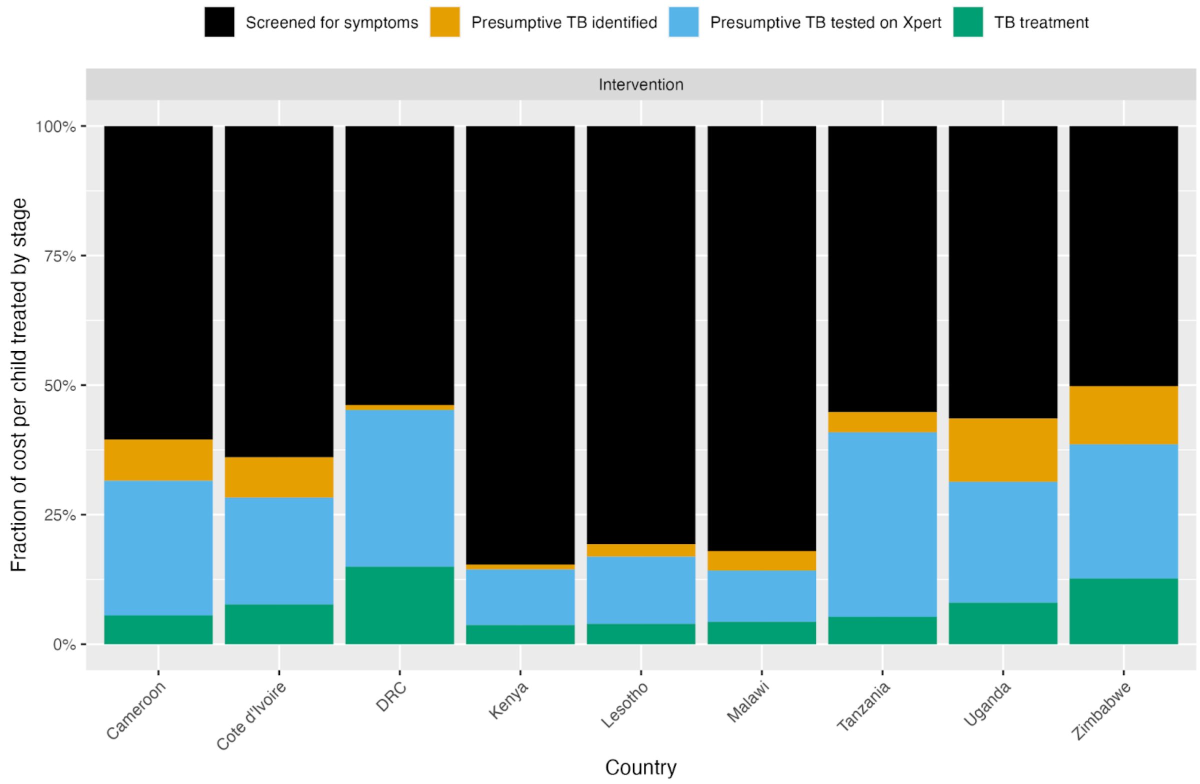

Figure A7 Composition of intervention costs for diagnosing and treating TB. DRC=Democratic Republic of the Congo, TB=tuberculosis.

The detailed disaggregation of children starting TPT by entry-point and age, as well as the mean number of households per household contact TPT initiation are shown in Table A56. Most countries had a roughly 1:1 ratio between index case households and children started on TPT, except Uganda where around 2 households were traced per child started on TPT. Very few household contacts 5 years or older were initiated on TPT, consistent with current priority targeting; conversely, the majority of children starting TPT at the HIV entry-point were 5 years or older. In all countries, more children started TPT as household contacts than via the HIV entry-point, but this varied from all, to a little over half. The cascade associated with screening of household contacts is shown in Table A67, aggregated across countries. Overall, around 2.5% of child contacts were started on ATT.

Table A5 The distribution of children initiated on TPT by age and entry point per country. DRC=Democratic Republic of the Congo, HIV=human immunodeficiency virus, TPT=tuberculosis preventive therapy.

country	% HIV-entry & <5 years	% HIV-entry & 5-14 years	% contact & <5 years	% contact & 5-14 years	households traced per contact starting PT
Cameroon	9.67	27.64	56.94	5.75	0.96
Côte d'Ivoire	0	0	93.48	6.52	1.14
DRC	1.03	3.64	95.08	0.26	0.94
Kenya	4.13	7.47	85.94	2.46	0.84
Lesotho	4.64	11.24	41.93	42.19	1.14
Malawi	10.2	19	68.38	2.41	1.19
Tanzania	13.67	22.46	62.23	1.64	0.66
Uganda	8.48	37.23	51.69	2.6	2.08
Zimbabwe	6.17	35.94	56.01	1.88	1.35

Table A6 Cascade for child case-finding during household contact screening

Quantity	Per 100 children screened under HHCM
Households screened	42.9
Children screened	100.0
Presumptive TB identified	9.7
Diagnosed TB	2.5

Modelled outcomes & health economic approach

Overview

Outcomes on mortality and incident cases averted through TPT are lacking from the data and were based on modelling. Models were stratified by age (0-4 years and 5-14 years), and HIV/ART status (assuming all children were on ART).

Changes in mortality were modelled by deriving a change in the fraction of children receiving ATT from the intervention effect analysis and applying estimates of case-fatality ratios from systematic review to the treated and untreated groups (stratified by age and HIV/ART status). The cascade of care, and associated resource use, leading up to a treatment was based on intervention data; the ratios of presumptive TB identified to ATT, and Xpert tests to ATT were assumed to be lower under standard of care by a factor based on comparing with baseline data (see Figure A8). The number of children screened was assumed proportional to presumptive TB identified; the ratio was based on data from the intervention in all countries, and data from some countries on how this ratio changed from before the intervention.

The change in children receiving TPT from the intervention effect analysis was disaggregated by entry-point (household contact or HIV entry-point). Different baseline risks of developing incident TB disease were assumed in these groups (ie household exposure vs background annual risk of infection, followed by progression). Hazard ratios for TPT effect from systematic review were applied to calculate the reduction in incidence. The probability of receiving ATT for incident TB was based on WHO age- and country-specific estimates of case detection ratio, scaled-up to account for likely superior detection among household contacts and HIV clinic attendees. Mortality was again modelled using relevant case-fatality ratios from literature for each group. To model the resources required, we used data on the number of households investigated per household contact starting TPT, and assumed that children starting PT via the HIV entry-point would receive screening comparable with that to identify presumptive TB.

Schematically, the health impact is based on the outcomes for those treated (ATT or PT) or not as

$$Impact = [RR \times treated + 0 \times untreated]$$
$$-[1 \times treated + (RR - 1) \times untreated]$$

and are thus calculated relative to each child treated at baseline.

Disability-adjusted life-years were calculated on the basis of life-years lost (i.e. neglecting decrement to quality of life during disease), discounted at 3%. Country-year and age specific UN Population Division World Population Prospects life tables were used to calculate a simple mean across ages within each age group (0-4 years and 5-14 years).

To quantify uncertainty we used probabilistic sensitivity analysis (PSA). Country-level effect estimates and baseline rates were drawn from the Markov chain Monte Carlo (MCMC) samples outputted from inference, and merged against samples of parameters used in the outcome modelling. 10,000 samples were used. Unit costs were applied to the activities driving cost included in the TIPPI data. We used gamma distributions to represent uncertainty in all SoC cost parameters used in the model. Uncertainty estimates for INT cost parameters were not available and not represented. The economic evaluation was performed from the public health system perspective, with outputs at

country-level including: incremental cost effectiveness ratio (US$ per DALY averted), and plots of the sampled results in the cost-effectiveness plane and cost-effectiveness acceptability curves. Results are presented for each country for the intervention as a whole, as well separately for preventive therapy and improved case detection.

Reproducibility and pre-registration

All code and data to reproduce this analysis are publicly available on GitHub https://github.com/petedodd/tippi. A health economic analysis plan (HEAP) was not produced for this study as it was first undertaken to inform a WHO guideline development plan under time constraints because the randomised studies within CaP-TB were delayed by the impact of the COVID-19 pandemic.

Details of modelling approach

Modelling resource use

For ATT, we considered: screening, testing using Xpert, starting TB treatment, and TB treatment success. Data on numbers screened was only available during the intervention. Comparable baseline data were not available for all quantities and countries. We therefore assumed that the ratio of children screened to presumptive TB identified was the same under the intervention as at baseline, and used data on the increased number of children with presumptive TB identified per child diagnosed with TB under intervention compared to baseline to reduce the baseline number of children screened per child started on ATT (see Figure A8). For countries lacking this data, we applied an average of this ratio across other countries. Similarly, we reduced the number of Xpert tests per child treated at baseline compared to under intervention using before/after estimates of its change, and averages across countries were lacking. Data on treatment success were not used in resource modelling: costs were assumed to apply for every child start on ATT.

For TPT, we used data in Table A56 and Table A67 to disaggregate activities by entry-point and to determine the number of households visited from the number of contacts starting TPT. As with ATT, costs for TPT were assumed to accrue regardless of completion of the course. To include household case-finding, resources were modelled for all countries using the aggregate data in Table A67.

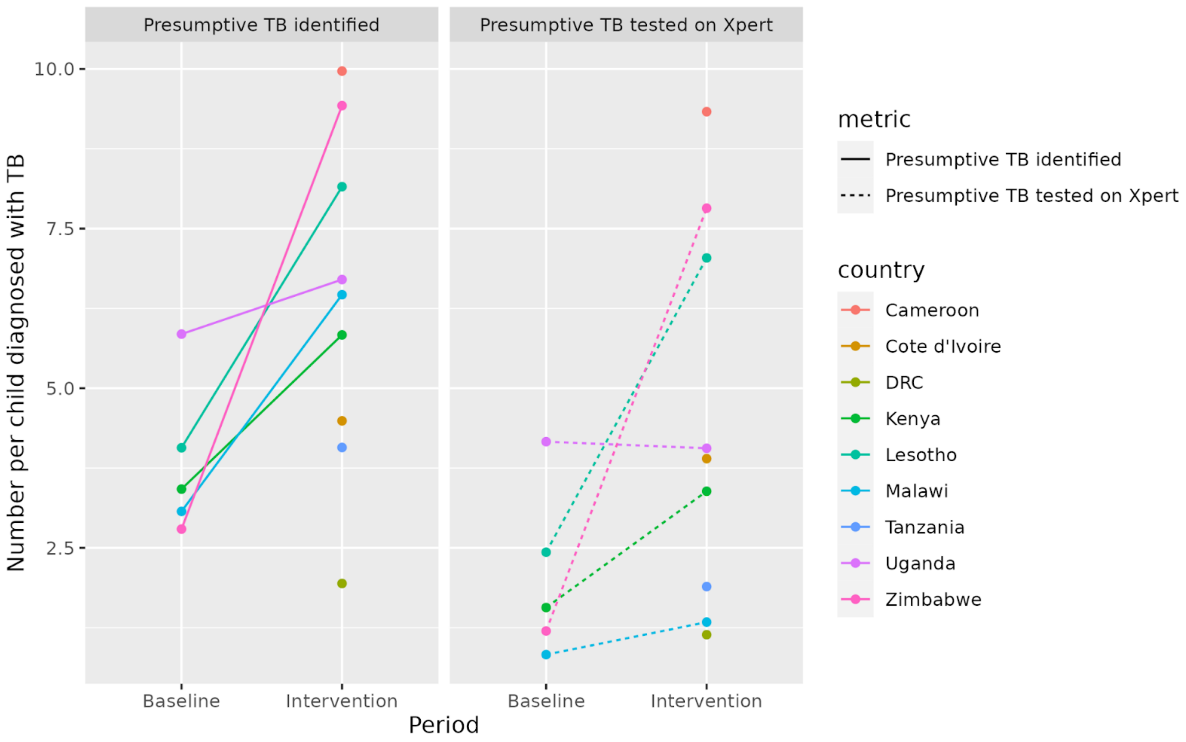

Figure A8 Change in resource use under intervention in comparison to the pre-intervention period (baseline). DRC=Democratic Republic of the Congo, TB=tuberculosis.

Modelling outcomes

Outcomes of TB with and without ATT were modelled using a case fatality ratio (CFR) approach, as followed in Dodd et al.[5] CFRs with and without ATT in each age group were based on the systematic review and meta-analysis of Jenkins et al.[6] The effect of HIV and ART on treatment outcomes was based on a analysis of Jenkins et al.[6] data presented in Dodd et al.[5] and untreated TB outcomes for children with HIV were based on the expert elicitation presented in Dodd et al.[5] All children with HIV were assumed to be on ART. Expected life-years and discounted life-years were based on simple means of interpolations of United Nations (World Population Prospects 2019 revision) country- and year-specific life tables described in Dodd et al.[7] using 2020 as the reference year (implemented using the discly R package available at https://github.com/petedodd/discly). The life-expectancy of children with HIV was not assumed to be the same as those without HIV-infection.

Because there are no child-specific tariffs for untreated TB disease, we used the adult tariff of 0.331 to capture the contribution of morbidity to DALYs.[8] While there are no data to directly inform the duration of TB disease with or without treatment in children, they are likely to be substantially shorter than the global average duration of disease for adults (~ 1 year). We therefore assumed that ultimately untreated TB in children had a mean duration of 6 months, and that children with ultimately treated TB had untreated TB for 3 months prior to treatment. As a sensitivity analysis to explore the importance of these assumptions, we also ran our analyses without including the morbidity contribution to DALYs.

For TPT, entry-point data shown in Table 6 was used to disaggregate those starting TPT by age and entry-point. All those starting PT through the HIV entry-point were assumed to be HIV-infected; HIV-infection prevalence in household contacts was based on data presented in Martinez et al.

(2017).[9] All children with HIV were assumed to be on ART. Incident TB risks for household contacts were base on combining systematic review data on LTBI prevalence from Fox et al,[10] with progression risks in those with LTBI from Martinez et al. (2020).[11] For children at the HIV entry-point, TB risk was based on a background community annual risk of TB infection (ARI), combined with progression risks base on Martinez et al. (2020).[11] Children without LTBI were assumed to have zero risk of incident TB. For children with HIV (ie all those at HIV entry-point and some household contacts), age-specific risks of incident TB had an incidence rate ratios from the systematic review of Dodd et al[12] applied to model the increased risk of TB due to HIV-infection, and the protection due to ART. The TPT hazard ratio from Martinez et al. (2020)[11] was applied to children modelled as having LTBI among household contacts, and an HIV-specific hazard ratio from Zunza et al[13] applied to children at the HIV entry-point. In the base case analysis, all children starting PT were assumed to receive full protection from review meta-analysis. In the 'TPT completion' sensitivity analysis, the proportion of children completing TPT under SoC and the intervention (Table A1) were assumed to receive protection, whereas the rest had no protection from TB incidence. PT was not assumed to have protective effects beyond the immediate risk of TB incidence.

The chances of incident TB being detected were based on WHO country- and age-specific estimates of case detection ratios (CDRs). Development of incident TB was assumed to happen over a 1 to 2 year timescale and discounting of health outcomes therefore neglected. TB outcomes with and without ATT were then modelled as described above. Implicitly a life-time horizon is used, with the assumption that differences in health outcomes and costs occur in the present. We neglected morbidity during TB.

Modelling and analysis was performed using R version 4.2.0.[14]

Parameter distributions

Parameter described in the previous sections were all represented in a probabilistic sensitivity analysis, sampling from the distributions shown in Table A78, which also shows the mean and interquartile range associated with each parameter.

Table A7 Parameters used in modelling outcomes. B = beta distribution; LN=log-normal distribution; MVN=multivariate normal distribution. IQR=interquartile range; OR=odds ratio; IRR=incidence rate ratio; HR=hazard ratio; RR=risk ratio; CFR=case fatality ratio; LTBI=latent TB infection; ARTI=annual risk of TB infection.

NAME	DISTRIBUTION	DESCRIPTION	SOURCE	MEAN (IQR)
**ontxY**	LN(-3.963316,0.6457913)	CFR children <5 on TB treatment	Jenkins et al 2017	0.019 (0.012 - 0.029)
**ontxO**	LN(-4.828314,0.4817445)	CFR children 5-14 on TB treatment	Jenkins et al 2017	0.008 (0.006 - 0.011)
**hivartOR:mn**	MVN: [2.6375681, -0.5683867]	ORs of death on TB treatment, (OR HIV+ vs -) x (ART -/+): mean	Jenkins et al 2017, Dodd et al 2017	
**hivartOR:sg**	MVN: [[0.2325509,-0.2325509],[-0.2325509,0.6367345]]	ORs of death on TB treatment, (OR HIV+ vs -) x (ART -/+): variance	Jenkins et al 2017, Dodd et al 2017	
**notxY**	LN(-0.830113,0.08035318)	CFR children <5 without TB treatment	Jenkins et al 2017	0.436 (0.413 - 0.460)
**notxO**	LN(-1.903809,0.1285165)	CFR children 5-14 without TB treatment	Jenkins et al 2017	0.149 (0.137 - 0.162)
**notxHAY**	B(15.18683,12.87500)	CFR children <5 without TB treatment (HIV+/ART+)	Dodd et al 2017	0.542 (0.478 - 0.605)
**notxHAO**	B(10.43383,11.08417)	CFR children 5-14 without TB treatment (HIV+/ART+)	Dodd et al 2017	0.484 (0.412 - 0.558)
**hivpi**	LN(2.066863,0.2800718)	IRR for TB incidence given HIV+/ART- (for individuals)	Dodd et al 2017	7.900 (6.540 - 9.543)
**artp**	LN(-1.203973,0.150482)	HR for TB incidence given HIV+/ART+ vs HIV+/ART-	Dodd et al 2016	0.300 (0.271 - 0.332)
**HHhivprev04**	B(55,526)	Prevalence of HIV in child HH contacts of HIV+ index case	Martinez et al 2017	0.094 (0.086 - 0.103)
**HHhivprev514**	B(54,854)	Prevalence of HIV in child HH contacts of HIV+ index case	Martinez et al 2017	0.059 (0.054 - 0.065)
**LTBI04**	B(106.7330582,193.9234438)	LTBI prevalence	Fox et al 2013	0.355 (0.336 - 0.373)
**LTBI514**	B(41.83776346,36.95275153)	LTBI prevalence	Fox et al 2013	0.531 (0.493 - 0.569)
**iptRRhivpos**	LN(-1.171183,0.5127492)	RR for incident TB given IPT in HIV+, age <15	Zunza et al 2017	0.310 (0.219 - 0.438)
**iptRRtstpos**	B(11.23657,113.6142)	RR for incident TB given IPT in TST+, age <15	Martinez et al 2020	0.090 (0.072 - 0.106)
**prog04**	B(5.152793,21.96717)	LTBI+ progression u5	Martinez et al 2020	0.182 (0.136 - 0.236)
**prog514**	B(4.151282,43.02238)	LTBI+ progression u5	Martinez et al 2020	0.082 (0.058 - 0.112)
**ari**	B(15.28457,3041.629)	ARTI per year	assumption	0.5% (0.31 - 0.64%)

Supplementary results

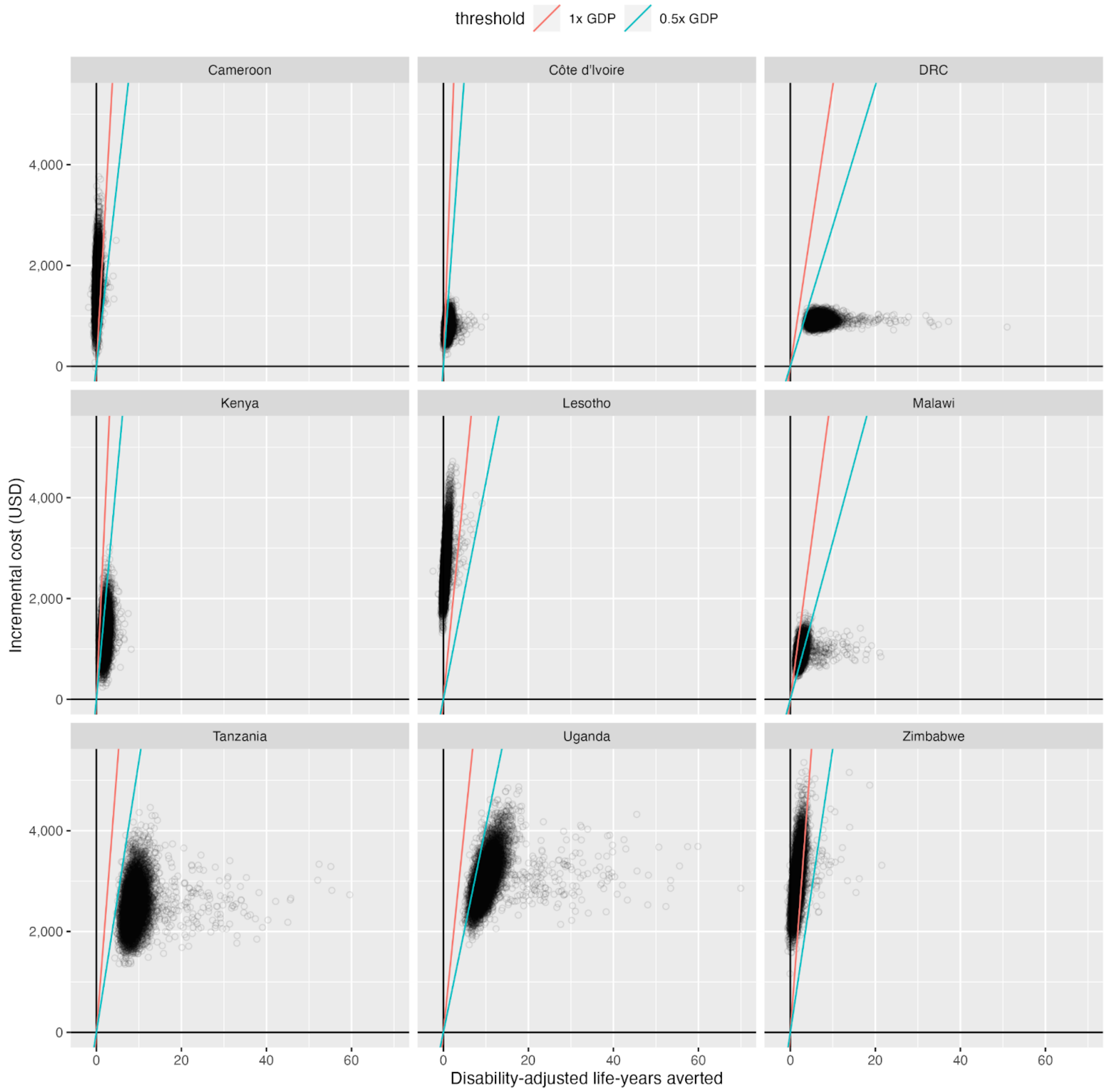

Figure A9 Cost-effectiveness plane showing the differences in costs (vertical axis) and disability-adjusted life-years (DALYs, horizontal axis) of implementing the combined Cap TB intervention package in each country, compared with standard of care from 1000 simulations. Threshold lines show the cost-effectiveness (willingness to pay) threshold based on 1X GDP per capita (red line) or 0.5X GDP per capita (green line) in each country. CaP-TB=Catalyzing Pediatric TB Innovations, DRC=Democratic Republic of the Congo, GDP=gross domestic product, TB=tuberculosis.

Table A8 Healthcare resource use, health outcomes, costs & cost-effectiveness of the Intensified case-finding intervention in comparison to standard of care (baseline) stratified by age. All outcomes are presented per 100 children initiating anti-tuberculosis treatment (ATT) or tuberculosis preventive therapy (TPT) at baseline. Data are presented as n (95% uncertainty interval) unless otherwise stated. All costs are presented in 2020 United States dollars ($USD). CaP-TB=Catalyzing Pediatric TB Innovations, DALY=disability-adjusted life years, DRC=Democratic Republic of the Congo, HIV=human immunodeficiency virus, SoC=standard of care, TB=tuberculosis, USD=United States dollar.

		**SoC (per 100 treated in SoC)**		**Intervention (per 100 treated in SoC)**		**Difference (per 100 treated in SoC)**					
Country	Age	TB treated	Cost	TB treated	Cost	TB treated	Cost	Deaths	Life-years (LYs)	Discounted LYs	**ICER ($/DALY)**
Cameroon	0-4	100	148,352 (61,895 to 270,733)	102 (78 to 131)	492,769 (278,277 to 787,196)	2 (-22 to 31)	344,417 (100,437 to 650,559)	-1 (-13 to 9)	46 (-603 to 861)	19 (-250 to 357)	18,257
Cameroon	5-14	100	149,073 (63,128 to 271,751)	99 (81 to 120)	478,205 (276,986 to 743,776)	-1 (-19 to 20)	329,132 (92,988 to 611,802)	0 (-4 to 4)	-16 (-251 to 252)	-7 (-110 to 111)	-45,452
Côte d'Ivoire	0-4	100	75,691 (33,459 to 136,556)	110 (77 to 154)	323,111 (184,805 to 513,237)	10 (-23 to 54)	247,420 (97,621 to 449,025)	-4 (-23 to 10)	262 (-596 to 1,418)	110 (-251 to 598)	2,242
Côte d'Ivoire	5-14	100	76,029 (33,195 to 137,254)	72 (56 to 92)	212,307 (132,040 to 319,280)	-28 (-44 to -8)	136,278 (37,741 to 251,746)	5 (1 to 8)	-283 (-483 to -72)	-127 (-217 to -32)	-1,071
DRC	0-4	100	13,698 (6,855 to 23,037)	148 (130 to 167)	77,937 (60,823 to 98,073)	48 (30 to 67)	64,239 (44,707 to 85,640)	-20 (-30 to -12)	1,264 (759 to 1,896)	521 (313 to 781)	123
DRC	5-14	100	13,664 (6,834 to 22,944)	180 (162 to 200)	95,142 (74,971 to 117,658)	80 (62 to 100)	81,478 (59,033 to 105,048)	-13 (-18 to -9)	783 (542 to 1,087)	341 (237 to 473)	239
Kenya	0-4	100	119,906 (49,618 to 218,344)	150 (121 to 185)	468,462 (263,803 to 746,524)	50 (21 to 85)	348,556 (122,551 to 635,239)	-21 (-36 to -8)	1,444 (584 to 2,504)	581 (235 to 1,008)	600
Kenya	5-14	100	120,578 (50,225 to 221,821)	117 (87 to 154)	363,939 (197,388 to 600,592)	17 (-13 to 54)	243,361 (51,275 to 488,607)	-2 (-8 to 2)	156 (-121 to 513)	66 (-51 to 218)	3,681
Lesotho	0-4	100	120,774 (52,416 to 217,087)	130 (85 to 190)	1,416,232 (897,340 to	30 (-15 to 90)	1,295,458 (768,592 to	-13 (-39 to 6)	780 (-375 to 2,415)	331 (-159 to 1,026)	3,911

					2,136,322)		2,019,077)				
Lesotho	5-14	100	121,302 (54,173 to 215,032)	55 (40 to 75)	603,716 (419,154 to 845,356)	-45 (-60 to -25)	482,414 (273,050 to 737,688)	9 (4 to 13)	-494 (-749 to -248)	-224 (-339 to -113)	-2,153
Malawi	0-4	100	54,082 (25,648 to 93,541)	257 (187 to 351)	503,191 (305,030 to 783,648)	157 (87 to 251)	449,109 (244,571 to 732,801)	-68 (-122 to -32)	4,713 (2,263 to 8,479)	1,887 (907 to 3,395)	238
Malawi	5-14	100	54,136 (25,492 to 93,485)	131 (105 to 162)	256,418 (161,291 to 375,689)	31 (5 to 62)	202,282 (100,013 to 326,733)	-7 (-15 to -1)	459 (81 to 980)	193 (34 to 411)	1,050
Tanzania	0-4	100	48,270 (22,385 to 84,516)	234 (196 to 277)	434,136 (283,606 to 623,059)	134 (96 to 177)	385,867 (231,851 to 577,072)	-57 (-83 to -37)	3,907 (2,522 to 5,698)	1,570 (1,014 to 2,291)	246
Tanzania	5-14	100	48,503 (21,907 to 83,908)	161 (130 to 198)	298,078 (193,156 to 434,883)	61 (30 to 98)	249,575 (138,744 to 387,219)	-11 (-20 to -5)	756 (349 to 1,290)	320 (148 to 545)	780
Uganda	0-4	100	62,271 (31,965 to 102,976)	297 (215 to 407)	531,243 (350,490 to 776,189)	197 (115 to 307)	468,971 (283,554 to 720,861)	-84 (-146 to -44)	5,710 (2,981 to 9,865)	2,317 (1,210 to 4,002)	202
Uganda	5-14	100	62,073 (31,929 to 101,714)	280 (199 to 390)	501,344 (327,798 to 737,873)	180 (99 to 290)	439,271 (262,346 to 677,650)	-37 (-65 to -18)	2,393 (1,148 to 4,156)	1,025 (493 to 1,778)	428
Zimbabwe	0-4	100	72,670 (31,949 to 127,993)	111 (84 to 147)	539,967 (334,522 to 813,437)	11 (-16 to 47)	467,297 (254,396 to 746,887)	-5 (-21 to 7)	319 (-454 to 1,373)	132 (-188 to 568)	3,537
Zimbabwe	5-14	100	72,130 (31,945 to 128,436)	100 (65 to 150)	485,311 (271,683 to 796,537)	0 (-35 to 50)	413,181 (191,580 to 732,371)	0 (-11 to 8)	3 (-467 to 659)	1 (-205 to 290)	302,979

Table A9 Healthcare resource use, health outcomes, costs & cost-effectiveness of the Household contact management & HIV clinic preventive therapy intervention in in comparison to standard of care (baseline) stratified by age. All outcomes are presented per 100 children initiating anti-tuberculosis treatment (ATT) or tuberculosis preventive therapy (TPT) at baseline. Data are presented as n (95% uncertainty interval) unless otherwise stated. All costs are presented in 2020 United States dollars ($USD). CaP-TB=Catalyzing Pediatric TB Innovations, DALY=disability-adjusted life years, DRC=Democratic Republic of the Congo, HIV=human immunodeficiency virus, SoC=standard of care, TB=tuberculosis, USD=United States dollar.

		**SoC (per 100 initiated in SoC)**		**Intervention (per 100 initiated in SoC)**		**Difference (per 100 initiated in SoC)**							
Country	Age	Started TPT	Cost	Started TPT	Cost	Started PT	Incident TB	ATT	TB deaths	Life-years (LYs)	Discounted LYs	Cost	**ICER ($/DALY)**
Cameroon	0-4	100	4,415 (1,974 to 8,169)	175 (158 to 193)	18,736 (14,184 to 25,482)	75 (58 to 93)	-4 (-9 to -2)	1 (0 to 2)	-2 (-5 to -1)	158 (73 to 295)	65 (30 to 123)	14,321 (11,888 to 17,770)	219
Cameroon	5-14	100	4,288 (1,858 to 8,012)	74 (55 to 99)	5,626 (3,377 to 9,185)	-26 (-45 to -1)	0 (0 to 1)	-1 (-1 to 0)	0 (0 to 0)	-15 (-29 to -1)	-7 (-13 to 0)	1,339 (-645 to 3,177)	-201
Côte d'Ivoire	0-4	100	3,792 (1,771 to 6,716)	229 (191 to 273)	35,234 (27,929 to 44,800)	129 (91 to 173)	-9 (-18 to -3)	5 (3 to 8)	-6 (-11 to -3)	349 (171 to 656)	147 (72 to 277)	31,442 (25,228 to 39,190)	213
Côte d'Ivoire	5-14	100	5,403 (2,550 to 9,743)	817 (587 to 1,136)	126,592 (87,947 to 180,929)	717 (487 to 1,036)	-33 (-76 to -9)	29 (13 to 47)	-16 (-26 to -9)	917 (538 to 1,529)	410 (240 to 686)	121,189 (84,094 to 173,865)	296
DRC	0-4	100	6,432 (2,301 to 14,591)	1,739 (1,508 to 2,008)	139,163 (111,079 to 178,382)	1,639 (1,408 to 1,908)	-113 (-219 to -40)	-11 (-68 to 24)	-49 (-95 to -21)	3,105 (1,324 to 6,066)	1,282 (546 to 2,505)	132,731 (105,567 to 170,204)	104
DRC	5-14	100	2,052 (862 to 3,890)	89 (73 to 108)	4,965 (3,606 to 6,909)	-11 (-27 to 8)	0 (0 to 0)	0 (0 to 0)	0 (0 to 0)	-5 (-13 to 4)	-2 (-6 to 2)	2,913 (1,958 to 3,966)	-1,356
Kenya	0-4	100	4,661 (2,122 to 8,477)	295 (249 to 349)	29,189 (21,586 to 40,190)	195 (149 to 249)	-13 (-26 to -4)	-5 (-16 to 2)	-4 (-9 to -1)	272 (73 to 635)	110 (30 to 256)	24,528 (18,599 to 32,933)	223
Kenya	5-14	100	3,526 (1,459 to 6,761)	32 (24 to 41)	1,789 (1,029 to 2,977)	-68 (-76 to -59)	1 (0 to 2)	-1 (-1 to 0)	0 (0 to 1)	-27 (-47 to -12)	-11 (-20 to -5)	-1,737 (-4,019 to -287)	151

Lesotho	0-4	100	3,219 (1,475 to 5,751)	221 (182 to 267)	17,623 (12,843 to 24,246)	121 (82 to 167)	-8 (-15 to -3)	4 (1 to 6)	-4 (-8 to -2)	274 (130 to 522)	116 (55 to 222)	14,404 (10,824 to 19,072)	124
Lesotho	5-14	100	3,298 (1,552 to 5,834)	305 (240 to 383)	23,467 (16,611 to 33,317)	205 (140 to 283)	-8 (-17 to -2)	8 (5 to 11)	-4 (-6 to -2)	217 (127 to 354)	98 (57 to 160)	20,168 (14,464 to 28,122)	206
Malawi	0-4	100	1,882 (800 to 3,520)	130 (115 to 146)	13,894 (11,752 to 16,614)	30 (15 to 46)	-2 (-4 to -1)	0 (-1 to 1)	-1 (-2 to 0)	63 (21 to 138)	25 (9 to 56)	12,013 (10,415 to 13,836)	476
Malawi	5-14	100	2,572 (1,171 to 4,552)	1,022 (662 to 1,536)	35,943 (20,234 to 61,537)	922 (562 to 1,436)	-5 (-13 to -1)	19 (10 to 33)	-9 (-16 to -4)	590 (297 to 1,051)	245 (124 to 437)	33,371 (18,550 to 57,599)	136
Tanzania	0-4	100	8,361 (2,384 to 21,385)	1,745 (1,311 to 2,321)	93,602 (58,939 to 148,380)	1,645 (1,211 to 2,221)	-94 (-190 to -32)	-35 (-126 to 19)	-30 (-76 to -5)	2,064 (352 to 5,243)	833 (145 to 2,111)	85,240 (50,977 to 138,158)	102
Tanzania	5-14	100	3,038 (1,374 to 5,599)	473 (366 to 605)	15,175 (7,279 to 28,280)	373 (266 to 505)	-1 (-3 to 0)	3 (0 to 6)	-1 (-3 to 0)	92 (23 to 185)	39 (10 to 77)	12,137 (5,765 to 22,994)	315
Uganda	0-4	100	3,427 (1,541 to 6,338)	367 (260 to 516)	172,309 (121,852 to 241,951)	267 (160 to 416)	-16 (-35 to -5)	2 (-10 to 12)	-8 (-18 to -3)	567 (210 to 1,202)	230 (85 to 488)	168,883 (119,096 to 237,203)	733
Uganda	5-14	100	4,723 (2,190 to 8,680)	1,291 (926 to 1,789)	103,561 (69,964 to 152,901)	1,191 (826 to 1,689)	-4 (-10 to -1)	37 (17 to 61)	-16 (-28 to -7)	1,002 (464 to 1,768)	426 (198 to 750)	98,839 (66,409 to 147,014)	232
Zimbabwe	0-4	100	3,739 (1,793 to 6,594)	158 (130 to 192)	61,154 (49,891 to 74,641)	58 (30 to 92)	-4 (-8 to -1)	-1 (-3 to 1)	-1 (-3 to 0)	91 (32 to 201)	38 (13 to 83)	57,415 (46,607 to 70,320)	1,522
Zimbabwe	5-14	100	8,882 (4,033 to 17,253)	2,933 (1,787 to 4,849)	307,261 (176,455 to 518,336)	2,833 (1,687 to 4,749)	-8 (-21 to -2)	45 (21 to 83)	-21 (-40 to -10)	1,280 (612 to 2,426)	559 (268 to 1,060)	298,379 (170,800 to 506,669)	534

Sensitivity analyses

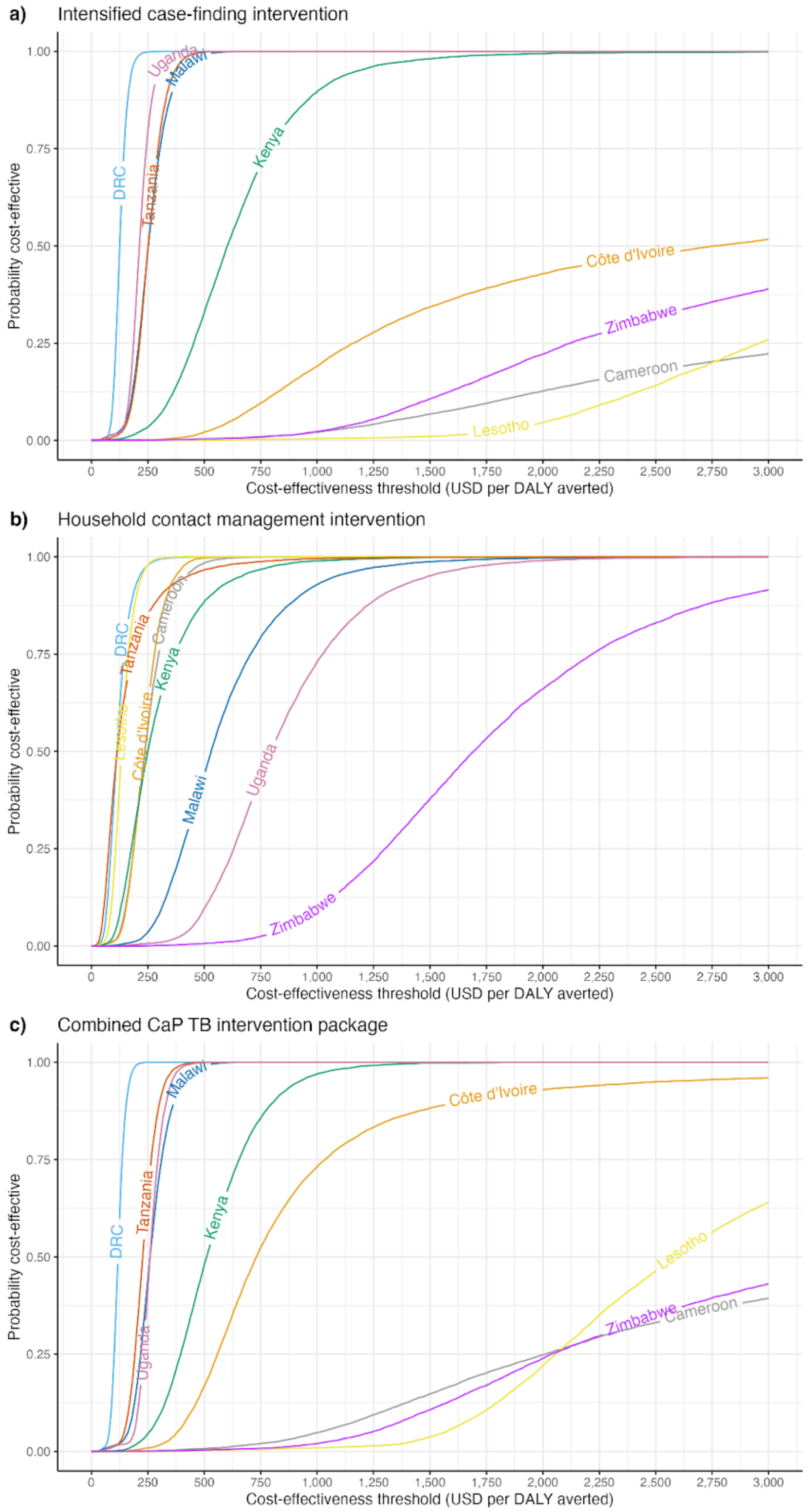

Figure A10 Cost-effectiveness acceptability curves for the Cap TB package of interventions in comparison to standard of care (age 0-4 years).

The figure shows the probability that an intervention is cost-effective in each country, based on the proportion of simulations in which the comparison of the intervention to the standard of care falls below the cost-effectiveness threshold shown on the horizontal axis. CaP-TB=Catalyzing Pediatric TB Innovations, DALY=disability-adjusted life years, DRC=Democratic Republic of the Congo, HIV=human immunodeficiency virus, USD=United States dollar.

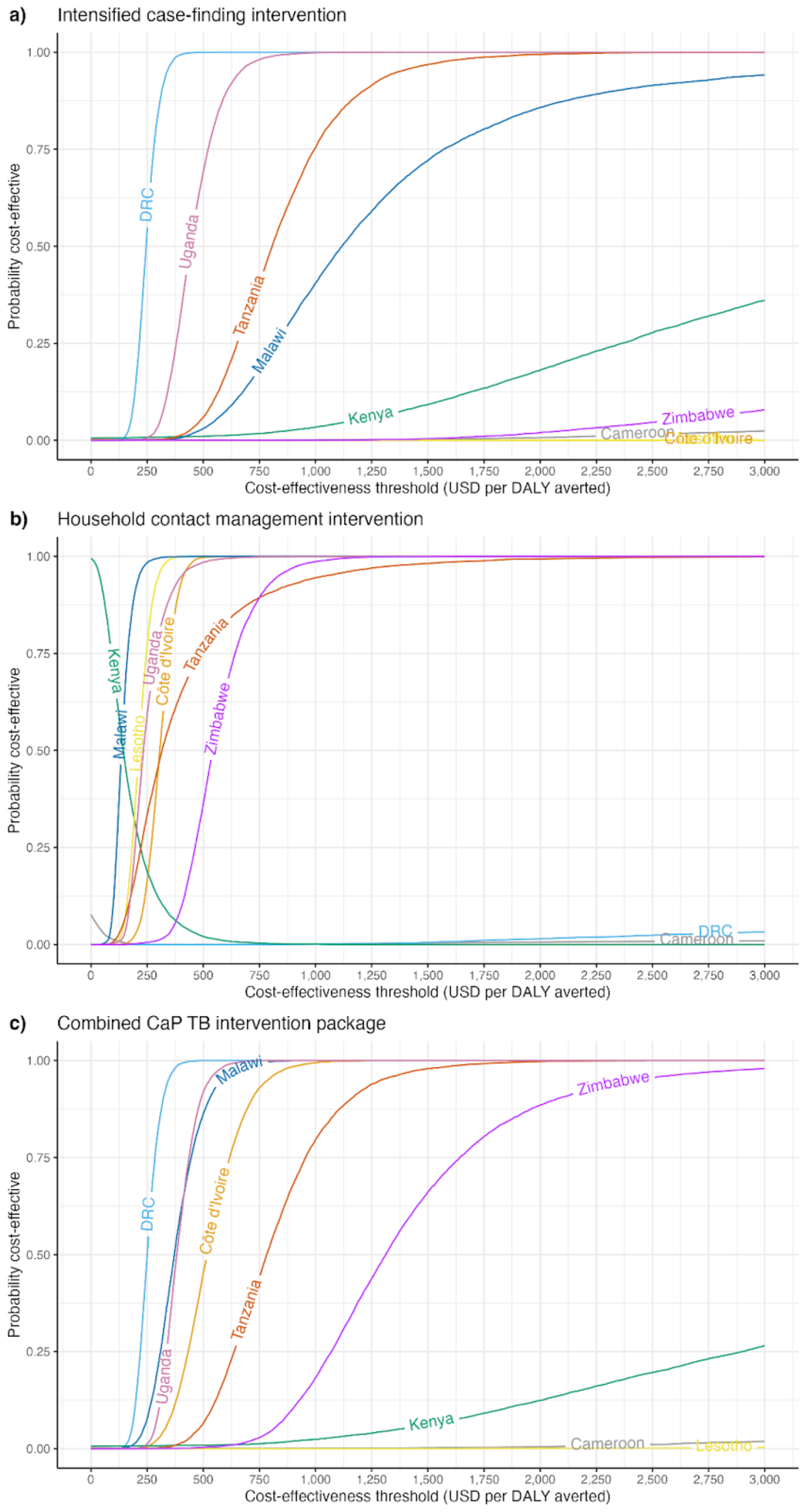

Figure A11 Cost-effectiveness acceptability curves for the Cap TB package of interventions in comparison to standard of care (age 5-14 years).

The figure shows the probability that an intervention is cost-effective in each country, based on the proportion of simulations in which the comparison of the intervention to the standard of care falls below the cost-effectiveness threshold shown on the horizontal axis. CaP-TB=Catalyzing Pediatric TB Innovations, DALY=disability-adjusted life years, DRC=Democratic Republic of the Congo, HIV=human immunodeficiency virus, USD=United States dollar.

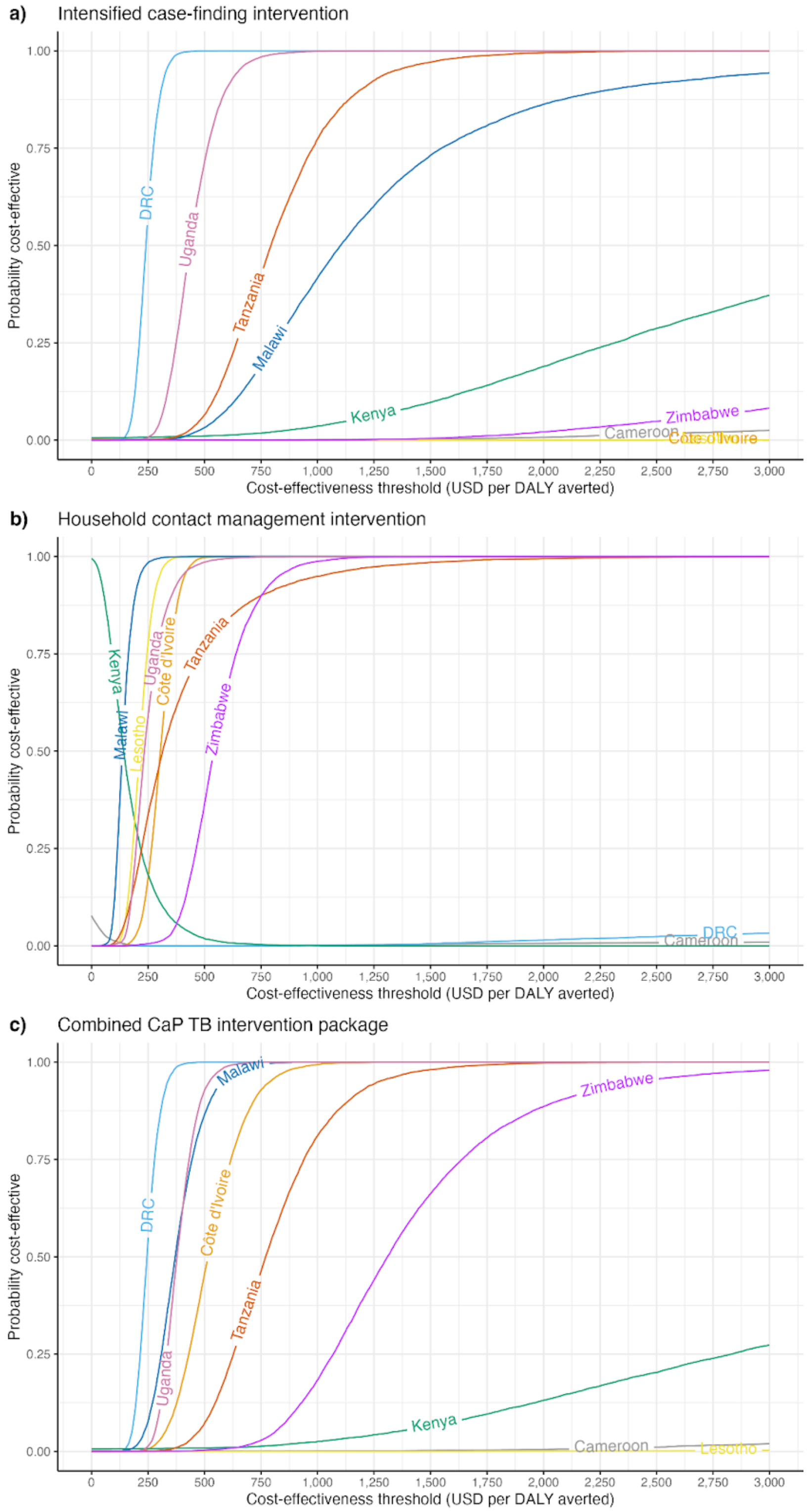

Figure A12 Cost-effectiveness acceptability curves for the combined Cap TB intervention package in comparison to standard of care with a discount rate of 0% per year. The figure shows the probability that an intervention is cost-effective in each country, based on the proportion of simulations in which the comparison of the intervention to the standard of care falls below the cost-effectiveness threshold shown on the horizontal axis. CaP-TB=Catalyzing Pediatric TB Innovations, DALY=disability-adjusted life years, DRC=Democratic Republic of the Congo, USD=United States dollar.

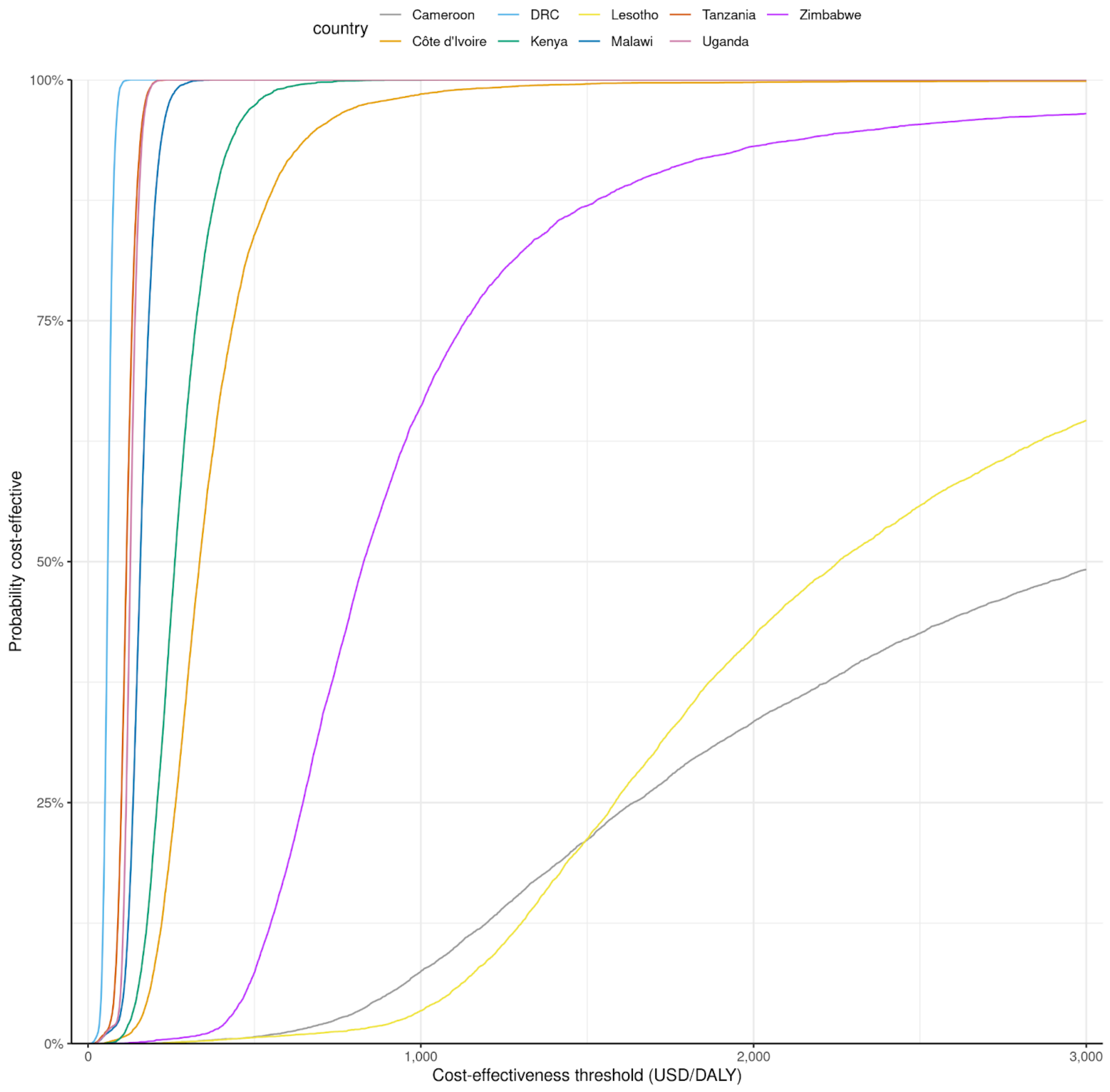

Figure A13 Cost-effectiveness acceptability curves for the combined Cap TB intervention package in comparison to standard of care with a discount rate of 5% per year. The figure shows the probability that an intervention is cost-effective in each country, based on the proportion of simulations in which the comparison of the intervention to the standard of care falls below the cost-effectiveness threshold shown on the horizontal axis. CaP-TB=Catalyzing Pediatric TB Innovations, DALY=disability-adjusted life years, DRC=Democratic Republic of the Congo, USD=United States dollar.

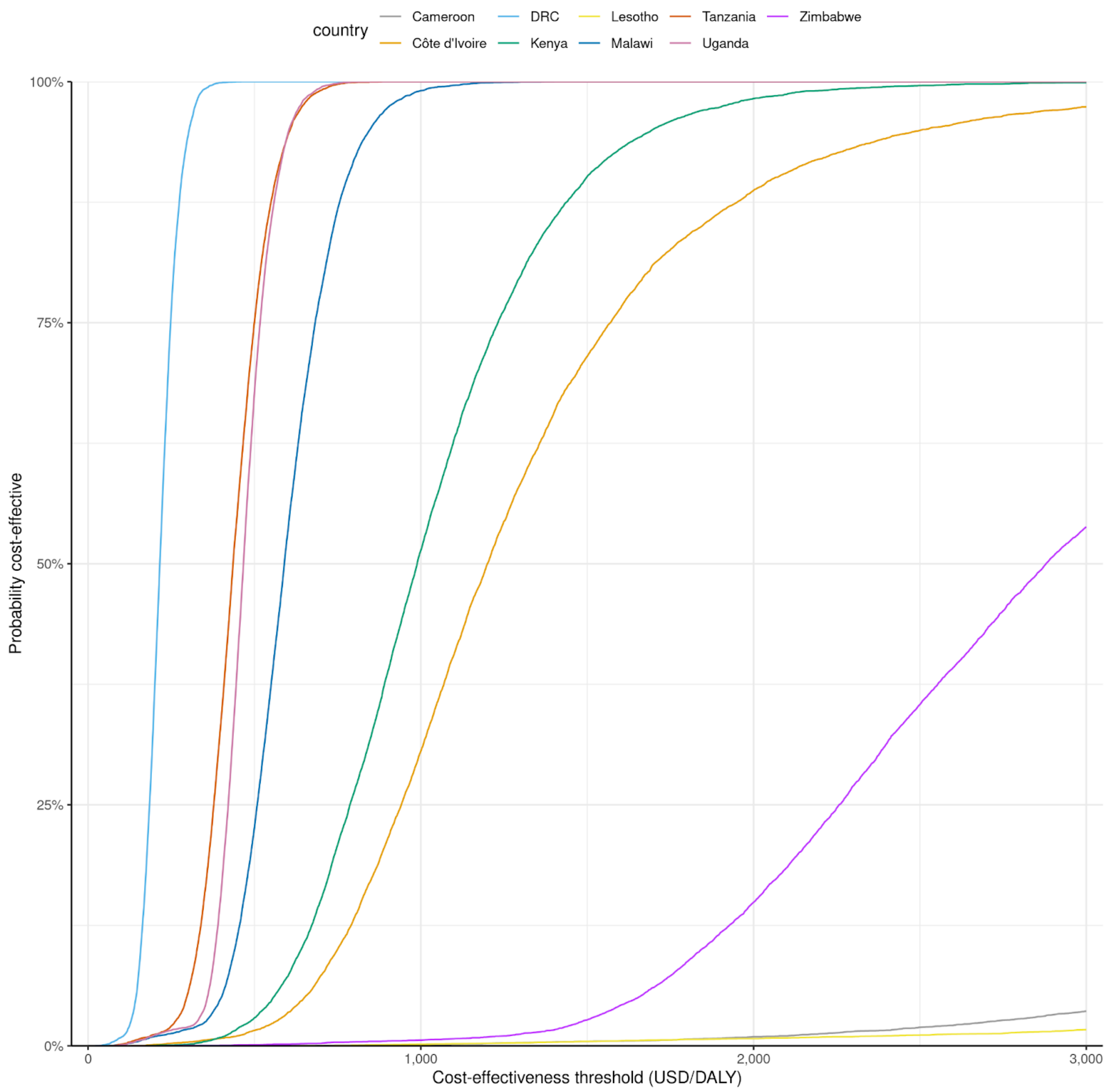

Figure A14 Cost-effectiveness acceptability curves for the combined Cap TB intervention package in comparison to standard of care with improvements in ATT success and TPT completion rates included. The figure shows the probability that an intervention is cost-effective in each country, based on the proportion of simulations in which the comparison of the intervention to the standard of care falls below the cost-effectiveness threshold shown on the horizontal axis. CaP-TB=Catalyzing Pediatric TB Innovations, DALY=disability-adjusted life years, DRC=Democratic Republic of the Congo, USD=United States dollar.

Table A10 Incremental cost-effectiveness ratios (ICERs) for different sensitivity analyses per country. ICERs are presented as cost in $US per discounted disability-adjusted life year (DALY) averted. ATT=anti-tuberculosis treatment, DRC=Democratic Republic of the Congo, TPT=tuberculosis preventive therapy. * ie DALYs without a contribution from morbidity

**country**	**Base case**	**Discounted life years***	**5% discount rate**	**0% discount rate**	**ATT/TPT completion improvement included**
Cameroon	6,804	6,862	10,370	2,809	1,346
Côte d'Ivoire	749	752	1,127	315	704
DRC	135	137	206	57	131
Kenya	634	640	975	257	573
Lesotho	4,949	4,935	7,426	2,046	4,392
Malawi	368	370	563	150	333
Tanzania	275	277	422	111	187
Uganda	295	298	450	122	278
Zimbabwe	1,819	1,826	2,721	782	822

Table A11 Cost-effectiveness threshold values where interventions first exceed 50% probability of being cost-effective (CET50) per country. The table does not show Cameroon and Lesotho because intervention does not exceed 50% probability of being cost-effective at all threshold values. DALY=disability-adjusted life year, DRC=Democratic Republic of the Congo, US$=United States dollar.

**Country**	**CET50 (US$/DALY averted)**
Côte d'Ivoire	799
DRC	144
Kenya	646
Malawi	387
Tanzania	285
Uganda	309
Zimbabwe	1,928

References

1. Gelman A, Hill J. Data Analysis Using Regression and Multilevel/Hierarchical Models. Cambridge University Press; 2006.

2. Goodrich B, Gabry J, Ali I, Brilleman S. rstanarm: Bayesian applied regression modeling via Stan. 2022. Available: https://mc-stan.org/rstanarm/

3. Official exchange rate (LCU per US$, period average). In: World Bank Open Data [Internet]. [cited 26 Jun 2023]. Available: https://data.worldbank.org/indicator/PA.NUS.FCRF

4. Turner HC, Lauer JA, Tran BX, Teerawattananon Y, Jit M. Adjusting for Inflation and Currency Changes Within Health Economic Studies. Value Health. 2019;22: 1026–1032.

5. Dodd PJ, Yuen CM, Sismanidis C, Seddon JA, Jenkins HE. The global burden of tuberculosis mortality in children: a mathematical modelling study. Lancet Glob Health. 2017;5: e898–e906.

6. Jenkins HE, Yuen CM, Rodriguez CA, Nathavitharana RR, McLaughlin MM, Donald P, et al. Mortality in children diagnosed with tuberculosis: a systematic review and meta-analysis. Lancet Infect Dis. 2017;17: 285–295.

7. Dodd PJ, Yuen CM, Jayasooriya SM, van der Zalm MM, Seddon JA. Quantifying the global number of tuberculosis survivors: a modelling study. Lancet Infect Dis. 2021. doi:10.1016/S1473-3099(20)30919-1

8. Salomon JA, Vos T, Hogan DR, Gagnon M, Naghavi M, Mokdad A, et al. Common values in assessing health outcomes from disease and injury: disability weights measurement study for the Global Burden of Disease Study 2010. Lancet. 2012;380: 2129–2143.

9. Martinez L, Shen Y, Handel A, Chakraburty S, Stein CM, Malone LL, et al. Effectiveness of WHO's pragmatic screening algorithm for child contacts of tuberculosis cases in resource-constrained settings: a prospective cohort study in Uganda. Lancet Respir Med. 2017. doi:10.1016/S2213-2600(17)30497-6

10. Fox GJ, Barry SE, Britton WJ, Marks GB. Contact investigation for tuberculosis: a systematic review and meta-analysis. Eur Respir J. 2013;41: 140–156.

11. Martinez L, Cords O, Robert Horsburgh C, Andrews JR, Acuna-Villaorduna C, Ahuja SD, et al. The risk of tuberculosis in children after close exposure: a systematic review and individual-participant meta-analysis. Lancet. 2020;395: 973–984.

12. Dodd PJ, Prendergast AJ, Beecroft C, Kampmann B, Seddon JA. The impact of HIV and antiretroviral therapy on TB risk in children: a systematic review and meta-analysis. Thorax. 2017;72: 559–575.

13. Zunza M, Gray DM, Young T, Cotton M, Zar HJ. Isoniazid for preventing tuberculosis in HIV-infected children. Cochrane Database Syst Rev. 2017;8: CD006418.

14. R Core Team. R: A language and environment for statistical computing. Vienna, Austria.: R Foundation for Statistical Computing; 2020. Available: https://www.R-project.org/
